# Deacetylated MDH1 and IDH1 aggravates PANoptosis in acute liver failure through endoplasmic reticulum stress signaling

**DOI:** 10.1038/s41420-024-02054-8

**Published:** 2024-06-08

**Authors:** Chunxia Shi, Yukun Wang, Jin Guo, Danmei Zhang, Yanqiong Zhang, Zuojiong Gong

**Affiliations:** Department of Infectious Diseases, Renmin Hospital of Wuhan University, Wuhan, China

**Keywords:** Hepatotoxicity, Cell death

## Abstract

Acute liver failure (ALF) is a disease with a high mortality rate and poor prognosis, whose pathogenesis is not fully understood. PANoptosis is a recently proposed mode of cell death characterized by pyroptosis, apoptosis, and necroptosis, but it cannot be explained by any of them alone. This study aims to explore the role of PANoptosis in ALF and the impact and mechanism of deacetylated malate dehydrogenase 1 (MDH1) and isocitrate dehydrogenase 1 (IDH1) on PANoptosis. Our results found that, compared with the control group, the cell viability in the lipopolysaccharide (LPS)/D-galactosamine (D-Gal) group decreased, lactate dehydrogenase (LDH) release increased, cell death increased, and the levels of PANoptosis-related molecules RIPK1, GSDMD, caspase-3, MLKL, IL-18, IL-1β increased, indicating that PANoptosis increased during ALF. Deacetylated MDH1 at K118 and IDH1 at K93 increased the expression of PANoptosis-related molecules RIPK1, GSDMD, caspase-3, MLKL, IL-18, and IL-1β in vivo and in vitro. The deacetylation weakened the inhibitory effect of histone deacetylase (HDAC) inhibitor ACY1215 on PANoptosis-related molecules, suggesting that deacetylated MDH1 at K118 and IDH1 at K93 aggravated PANoptosis during ALF. Deacetylated MDH1 at K118 and IDH1 at K93 also promoted the expression of endoplasmic reticulum stress-related molecules BIP, ATF6, XBP1, and CHOP in vivo and in vitro. The use of endoplasmic reticulum stress inhibitor 4-PBA weakened the promotion effect of deacetylated MDH1 K118 and IDH1 K93 on PANoptosis. The results suggested that deacetylated MDH1 at K118 and IDH1 at K93 may aggravate PANoptosis in ALF through endoplasmic reticulum stress signaling. In conclusion, deacetylated MDH1 and IDH1 may aggravate PANoptosis in ALF, and the mechanism may act through endoplasmic reticulum stress signaling.

## Introduction

Acute liver failure (ALF) is defined as severe liver dysfunction characterized by an international normalized ratio (INR) of ≥1.5 and altered mental status caused by hepatic encephalopathy in patients with no known liver diseases. The interval between the onset of jaundice and the development of hepatic encephalopathy is usually no more than 26 weeks [1, 2]. ALF has a high mortality rate, leading to significant health and economic burdens worldwide. At present, there is still a lack of effective treatments for ALF [3]. Liver transplantation is the only recommended treatment for ALF. However, liver transplantation is limited by the scarcity of liver transplant resources and organ rejection. Therefore, an in-depth study of the pathophysiological mechanism of ALF may provide new strategies for the clinical treatment of ALF.

Recent studies have reported that pyroptosis, apoptosis, and necroptosis interact with each other. Therefore, a concept of total cell death has been proposed, called PANoptosis, which is characterized by pyroptosis, apoptosis, and necroptosis, but cannot be explained by any of them alone [4]. PANoptosis is regulated by a cascade of molecular signals. The molecules assemble into complexes called PANoptosome. PANoptosis is associated with a variety of diseases, including infectious and oncological diseases [5, 6]. Pyroptosis includes classical signaling pathways mediated by caspase-1 and non-classical signaling pathways mediated by caspase-4, 5, and 11 [7]. The inflammasome binds to caspase-1 and activates it. Activated caspase-1 cleaves interleukin-1β (IL-1β) and IL-18 precursors, facilitating the secretion of IL-1β and IL-18 and inducing cell death [8]. The non-classical pyroptosis pathway is mainly mediated by caspase-4, caspase-5, and caspase-11. After the activation of caspase-4, caspase-5, and caspase-11, Gasdermin-D (GSDMD) is cleaved to initiate pyroptosis [9]. Apoptosis involves both endogenous and exogenous pathways. The endogenous apoptosis pathway is also known as the mitochondrial apoptosis pathway. Apoptosis is characterized by the activation of caspases, nucleus fragmentation, and the formation of apoptotic bodies. Caspase-3 plays a crucial role in apoptosis. Necroptosis is mainly regulated by receptor-interacting serine/threonine-protein kinase 1 (RIPK1) and mixed lineage kinase domain-like (MLKL). RIPK1 activates the autophosphorylation of RIPK3, which in turn activates MLKL, leading to cell rupture and ultimately cell death [10].

The liver is the major organ related to energy metabolism. Hepatocytes are rich in mitochondria, which serve as the main site of metabolism, producing adenosine triphosphate (ATP) to supply energy for the normal functioning of the liver. Many pathogenic factors in the liver, such as viral infections, drugs, and inflammation, can induce mitochondrial damage and dysfunction by interfering with the tricarboxylic acid cycle [11]. Therefore, in the case of liver failure, the oxygen consumption of liver cells is decreased, the expression of genes related to the tricarboxylic acid cycle is reduced, and the energy metabolism is impaired [12, 13]. In our previous study, we utilized proteomics and discovered that the expression of energy metabolic enzymes malate dehydrogenase 1 (MDH1) and isocitrate dehydrogenase 1 (IDH1) decreased in ALF [14]. Subsequent studies revealed that the histone deacetylase (HDAC) inhibitor ACY1215 alleviated ALF by modulating acetylation at MDH1 K118 and IDH1 K93 sites. However, the role of PANoptosis in ALF and its relationship with MDH1 and IDH1 acetylation remain unclear. Therefore, in this study, we explored the role of PANoptosis in ALF and the effects and mechanisms of deacetylated MDH1 and IDH1 on PANoptosis. This research aims to provide theoretical basis for the clinical treatment of ALF.

## Results

### PANoptosis was increased in LPS/D-Gal-induced cell injury

**Fig. 1:**
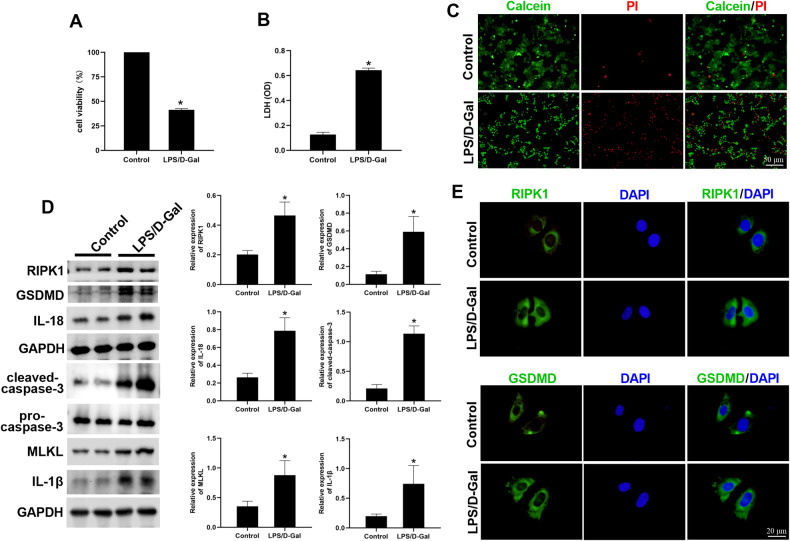
PANoptosis was increased in LPS/D-Gal induced hepatocyte injury. **A** Cell viability, (**B**) LDH release, and (**C**) PI staining of AML-12 cells in each group. **D** The expression of PANoptosis-related molecules RIPK1, GSDMD, caspase-3, MLKL, IL-18, and IL-1β in AML-12 cells was detected by Western blot. **E** The expression of RIPK1 and GSDMD in AML-12 cells was detected by immunofluorescence. The results are presented as mean ± SD based on three repetitions. * compared with the control group, *P* < 0.05.

We first assessed the level of PANoptosis during the ALF process. After the cells were stimulated with LPS/D-Gal, cell death and the expression of PANoptosis-related molecules were detected. As shown in Fig. 1A–C, cell viability decreased, lactate dehydrogenase (LDH) release increased, and propyl iodide (PI) staining indicated increased cell death in the LPS/D-Gal group compared with the control group. The levels of PANoptosis-related molecules RIPK1, GSDMD, caspase-3, MLKL, IL-18, and IL-1β were increased in the LPS/D-Gal group compared with the control group (Fig. 1D, E), indicating elevated levels of PANoptosis in ALF.

### Deacetylated MDH1 and IDH1 aggravated PANoptosis during ALF

**Fig. 2:**
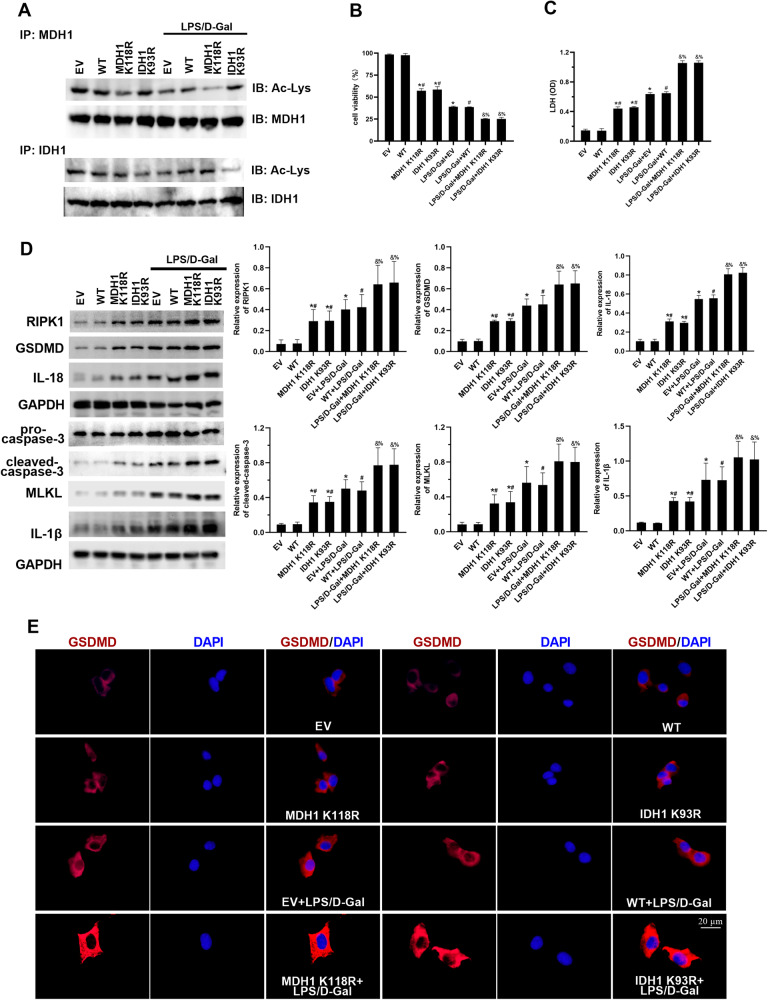
Deacetylated MDH1 and IDH1 aggravated PANoptosis during ALF. **A** The levels of MDH1 and IDH1 acetylation, (**B**) cell viability and (**C**) LDH release of AML-12 cells in each group. **D** The expression of PANoptosis-related molecules RIPK1, GSDMD, caspase-3, MLKL, IL-18 and IL-1β in AML-12 cells was detected by Western blot. **E** The expression of GSDMD in AML-12 cells was detected by immunofluorescence. The results are presented as mean ± SD based on three repetitions. * compared with EV group, *P* < 0.05; # compared with WT group, *P* < 0.05; & compared with LPS/D-Gal + EV group, *P* < 0.05; % compared with LPS/D-Gal + WT group, *P* < 0.05.

In our previous studies, we found that the acetylation of MDH1 K118 and IDH1 K93 is closely related to the activity and function of MDH1 and IDH1 [15, 16]. Therefore, in this study, we mutated the MDH1 K118 and IDH1 K93 sites (lysine K mutated to arginine R) to simulate deacetylation. We examined the impact of deacetylated MDH1 and IDH1 on PANoptosis in ALF. The results showed that compared with the LPS/D-Gal + empty vector (EV) and LPS/D-Gal + wild-type (WT) groups, the acetylation levels of MDH1 or IDH1 in LPS/D-Gal + MDH1 K118R or LPS/D-Gal + IDH1 K93R group were significantly decreased. Additionally, cell viability decreased, and LDH release increased in the LPS/D-Gal + MDH1 K118R and LPS/D-Gal + IDH1 K93R groups compared with the LPS/D-Gal +EV and LPS/D-Gal + WT groups (Fig. 2A–C). Moreover, the expressions of PANoptosis-related molecules RIPK1, GSDMD, caspase-3, MLKL, IL-18, and IL-1β were increased in the LPS/D-Gal + MDH1 K118R and LPS/D-Gal + IDH1 K93R groups compared with the LPS/D-Gal +EV and LPS/D-Gal + WT groups (Fig. 2D, E). These results indicated that deacetylated MDH1 and IDH1 aggravated PANoptosis in ALF.

### Deacetylated MDH1 and IDH1 weakened the inhibitory effect of the HDAC inhibitor ACY1215 on PANoptosis

**Fig. 3:**
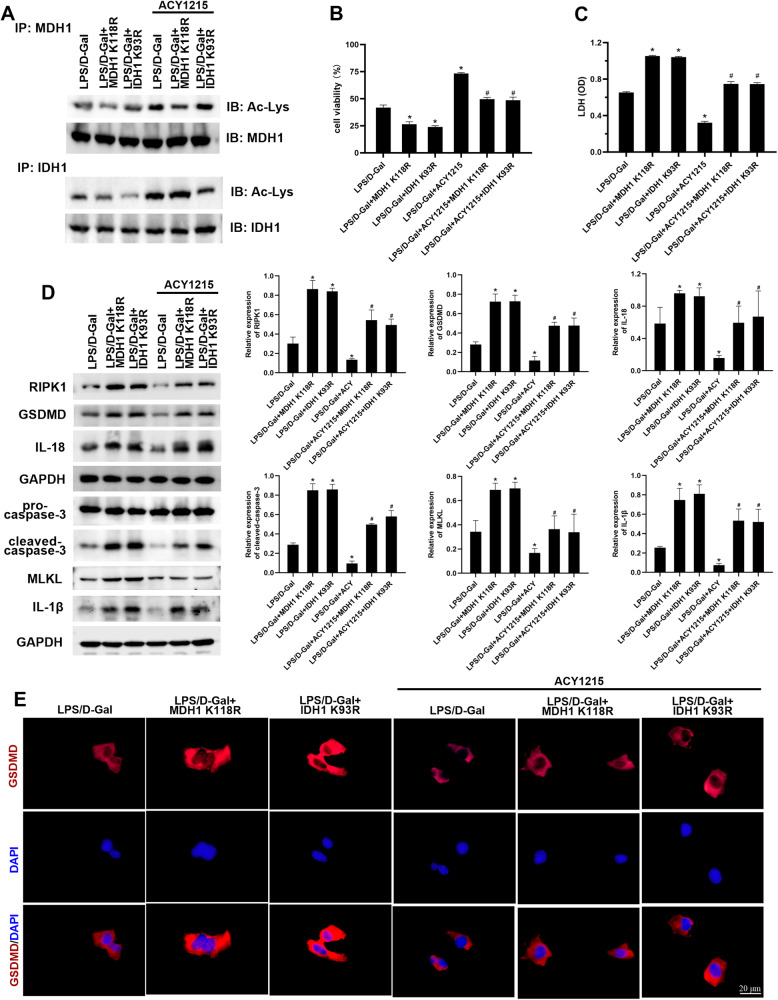
Deacetylated MDH1 and IDH1 weakened the inhibition of HDAC inhibitor ACY1215 on PANoptosis. **A** The levels of MDH1 and IDH1 acetylation, (**B**) cell viability, and (**C**) LDH release of AML-12 cells in each group. **D** The expression of PANoptosis-related molecules RIPK1, GSDMD, caspase-3, MLKL, IL-18 and IL-1β in AML-12 cells was detected by Western blot. **E** The expression of GSDMD in AML-12 cells was detected by immunofluorescence. The results are presented as mean ± SD based on three repetitions. * compared with LPS/D-Gal group, *P* < 0.05; # compared with LPS/D-Gal + ACY1215 group, *P* < 0.05.

In order to further clarify the effect of deacetylated MDH1 and IDH1 on PANoptosis, the HDAC inhibitor ACY1215 was used. The results showed that compared with the LPS/D-Gal group, the acetylation levels of MDH1 or IDH1 in LPS/D-Gal + ACY1215 group were increased. Cell viability was increased, and LDH release was decreased in LPS/D-Gal + ACY1215 group compared with the LPS/D-Gal group. Besides, compared with the LPS/D-Gal + ACY1215 group, the acetylation levels of MDH1 or IDH1 in the LPS/D-Gal + ACY1215 + MDH1 K118R group or LPS/D-Gal + ACY1215 + IDH1 K93R group were decreased. Additionally, cell viability decreased, and LDH release increased in the LPS/D-Gal + ACY1215 + MDH1 K118R group and LPS/D-Gal + ACY1215 + IDH1 K93R group compared with the LPS/D-Gal + ACY1215 group (Fig. 3A–C). We further examined the levels of PANoptosis and found that compared with the LPS/D-Gal group, the levels of PANoptosis-related molecules RIPK1, GSDMD, caspase-3, MLKL, IL-18, and IL-1β were decreased in the LPS/D-Gal + ACY1215 group. Compared with LPS/D-Gal + ACY1215 group, the levels of PANoptosis-related molecules RIPK1, GSDMD, caspase-3, MLKL, IL-18, and IL-1β were increased in LPS/D-Gal + ACY1215 + MDH1 K118R group and LPS/D-Gal + ACY1215 + IDH1 K93R group (Fig. 3D, E). These results indicated that deacetylated MDH1 and IDH1 weakened the inhibitory effect of the HDAC inhibitor ACY1215 on PANoptosis, suggesting that the acetylation of MDH1 and IDH1 influenced PANoptosis.

### Deacetylated MDH1 and IDH1 could exacerbate liver injury and aggravate PANoptosis in liver tissues of mice with ALF

**Fig. 4:**
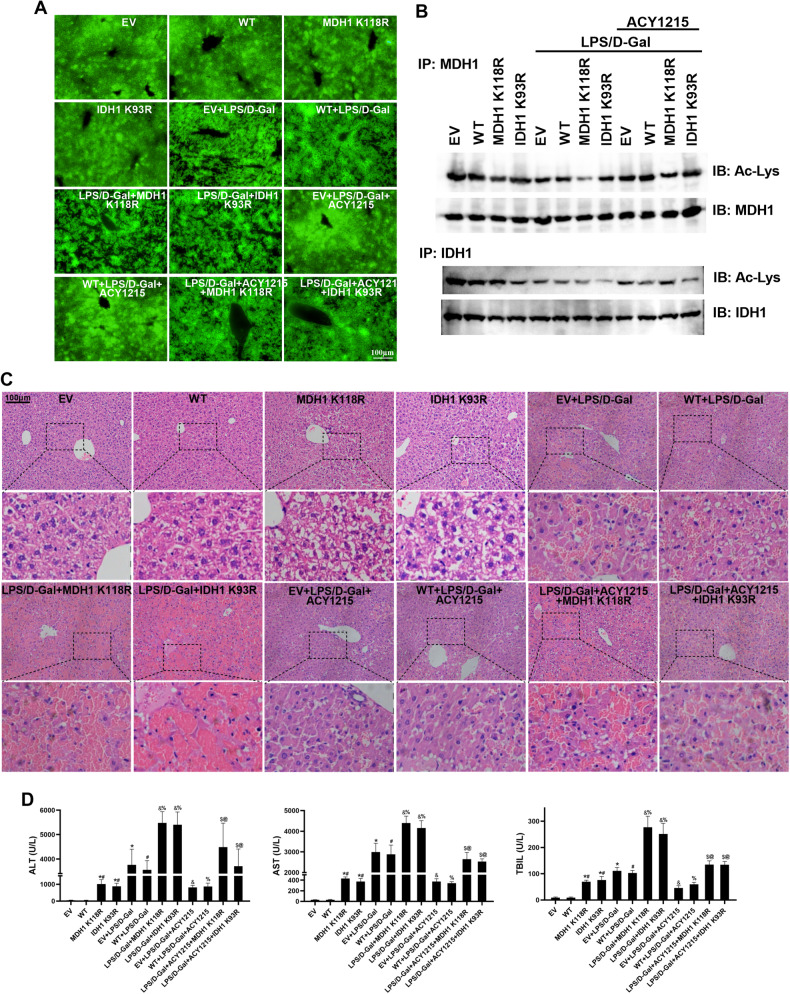
Deacetylated MDH1 and IDH1 aggravated liver tissue injury in mice. **A** Representative fluorescent images of adeno-associated viruses and (**B**) acetylation levels of MDH1 and IDH1 in liver tissues of mice in each group. **C** HE staining of mice liver tissues in each group. **D** Serum ALT, AST, and TBIL levels of mice in each group. The results are presented as mean ± SD based on three repetitions. * compared with EV group, *P* < 0.05; # compared with WT group, *P* < 0.05; & compared with EV + LPS/D-Gal group, *P* < 0.05; % compared with WT + LPS/D-Gal group, *P* < 0.05; $ compared with EV + LPS/D-Gal + ACY1215 group, *P* < 0.05; @ compared with WT + LPS/D-Gal + ACY1215 group, *P* < 0.05.

**Fig. 5:**
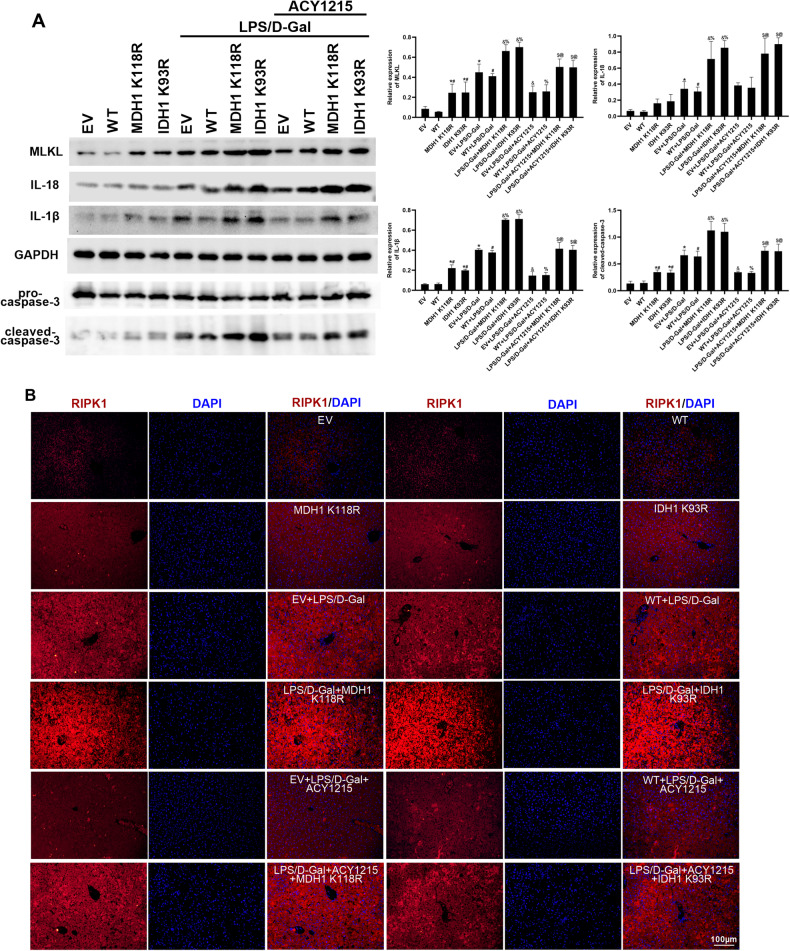
Deacetylated MDH1 and IDH1 aggravated PANoptosis in liver tissues of ALF mice. **A** The expression of PANoptosis-related molecules MLKL, caspase-3, IL-18, and IL-1β in mice liver tissues was detected by Western blot. **B** The expression of RIPK1 in mice liver tissues was detected by immunofluorescence. The results are presented as mean ± SD based on three repetitions. * compared with EV group, *P* < 0.05; # compared with WT group, *P* < 0.05; & compared with EV + LPS/D-Gal group, *P* < 0.05; % compared with WT + LPS/D-Gal group, *P* < 0.05; $ compared with EV + LPS/D-Gal + ACY1215 group, *P* < 0.05; @ compared with WT + LPS/D-Gal + ACY1215 group, *P* < 0.05.

We further verified these results in mice. MDH1 K118R and IDH1 K93R mutated adeno-associated viruses were injected into mice and the HDAC inhibitor ACY1215 was administered in mice. As shown in Fig. 4A, B, the MDH1 K118R or IDH1 K93R mutations significantly reduced the acetylation levels of MDH1 or IDH1. HE staining and liver function test results showed that liver injury was more severe, and the levels of alanine aminotransferase (ALT), aspartate aminotransferase (AST), and total bilirubin (TBIL) were increased in the LPS/D-Gal + MDH1 K118R group and LPS/D-Gal + IDH1 K93R group compared with the LPS/D-Gal + EV and LPS/D-Gal + WT group. In addition, compared with the LPS/D-Gal + ACY1215 group, liver damage increased, and the levels of ALT, AST, and TBIL were elevated in the LPS/D-Gal + ACY1215 + MDH1 K118R group and LPS/D-Gal + ACY1215 + IDH1 K93R group (Fig. 4C, D). These results indicated that deacetylated MDH1 and IDH1 could exacerbate liver tissue damage in mice with ALF. We further examined the levels of PANoptosis in the liver tissues of mice. The results showed that compared with the LPS/D-Gal + EV and LPS/D-Gal + WT groups, the levels of PANoptosis-related molecules RIPK1, caspase-3, MLKL, IL-18, and IL-1β were increased in the LPS/D-Gal + MDH1 K118R group and LPS/D-Gal + IDH1 K93R group. Besides, compared with LPS/D-Gal + ACY1215 group, the levels of these molecules in LPS/D-Gal + ACY1215 + MDH1 K118R and LPS/D-Gal + ACY1215 + IDH1 K93R groups were significantly increased (Fig. 5A, B). These results suggested that deacetylated MDH1 and IDH1 could elevate the level of PANoptosis in the liver tissues of ALF mice.

### Deacetylated MDH1 and IDH1 promoted endoplasmic reticulum stress signaling in vivo and in vitro

**Fig. 6:**
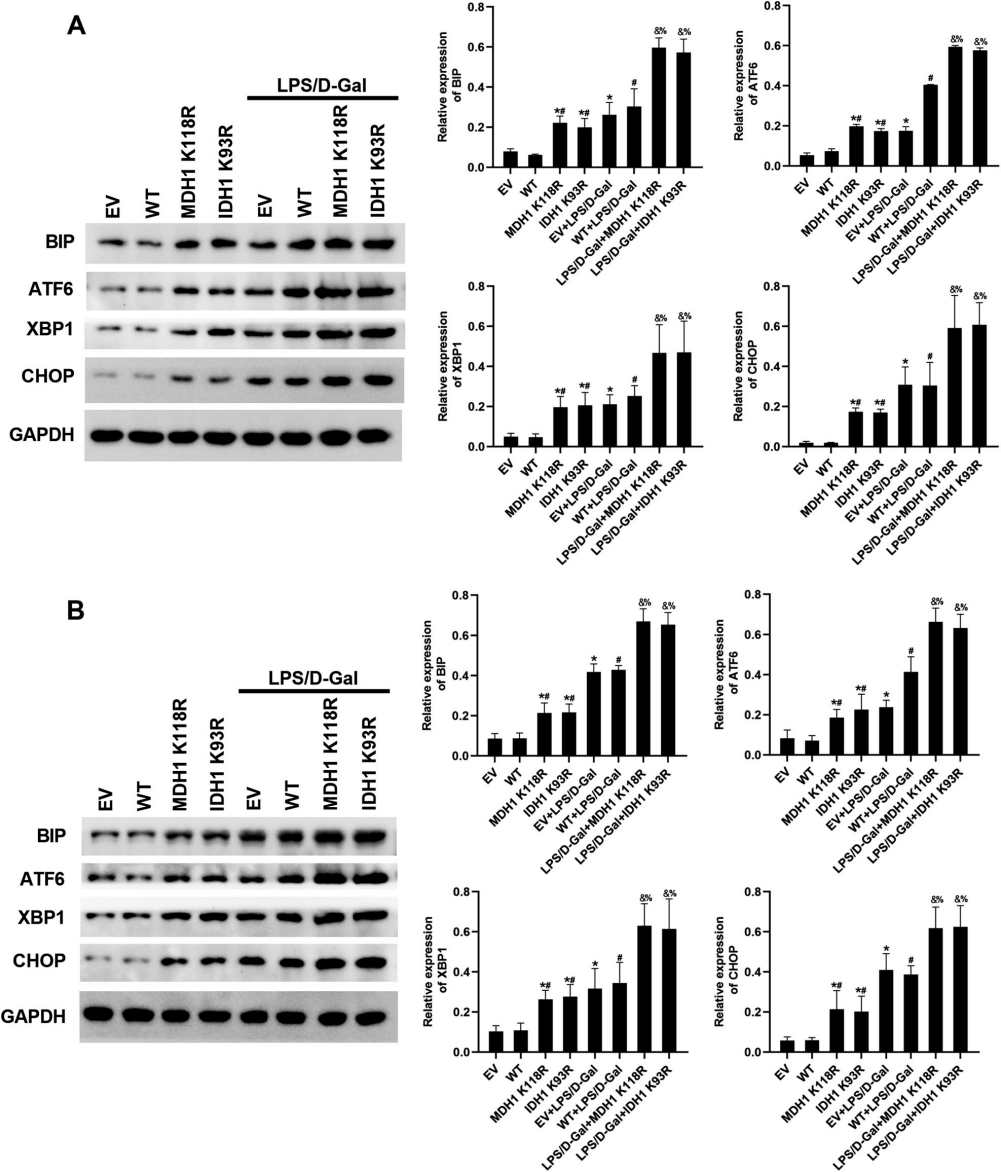
Deacetylated MDH1 and IDH1 promoted ER stress signaling in vivo and in vitro. **A** The expression of ER stress-related molecules BIP, ATF6, XBP1, CHOP in AML-12 cells was detected by Western blot. **B** The expression of ER stress-related molecules BIP, ATF6, XBP1, CHOP in mice liver tissues was detected by Western blot. The results are presented as mean ± SD based on three repetitions. * compared with EV group, *P* < 0.05; # compared with WT group, *P* < 0.05; & compared with EV + LPS/D-Gal group, *P* < 0.05; % compared with WT + LPS/D-Gal group, *P* < 0.05.

Deacetylated MDH1 and IDH1 deacetylation could aggravate PANoptosis during ALF, but the mechanism is unclear. Studies have shown that endoplasmic reticulum (ER) stress is associated with the occurrence and development of a variety of liver diseases, such as non-alcoholic fatty liver disease, alcohol-related liver disease, viral hepatitis, liver ischemia, and liver cancer [17]. Therefore, we examined the effects of deacetylated MDH1 and IDH1 on ER stress. Results of cell and animal experiments showed that compared with LPS/D-Gal + EV and LPS/D-Gal + WT groups, the expression of ER stress-related molecules activating transcription factor 6 (ATF6), heavy-chain binding protein (BIP), X-box binding protein 1 (XBP1), C/EBP-homologous protein (CHOP) were increased in LPS/D-Gal + MDH1 K118R and LPS/D-Gal + IDH1 K93R groups (Fig. 6A, B). The results suggested that deacetylated MDH1 and IDH1 could promote ER stress signaling in vivo and in vitro.

### Deacetylated MDH1 and IDH1 aggravated PANoptosis during ALF through ER stress signaling

**Fig. 7:**
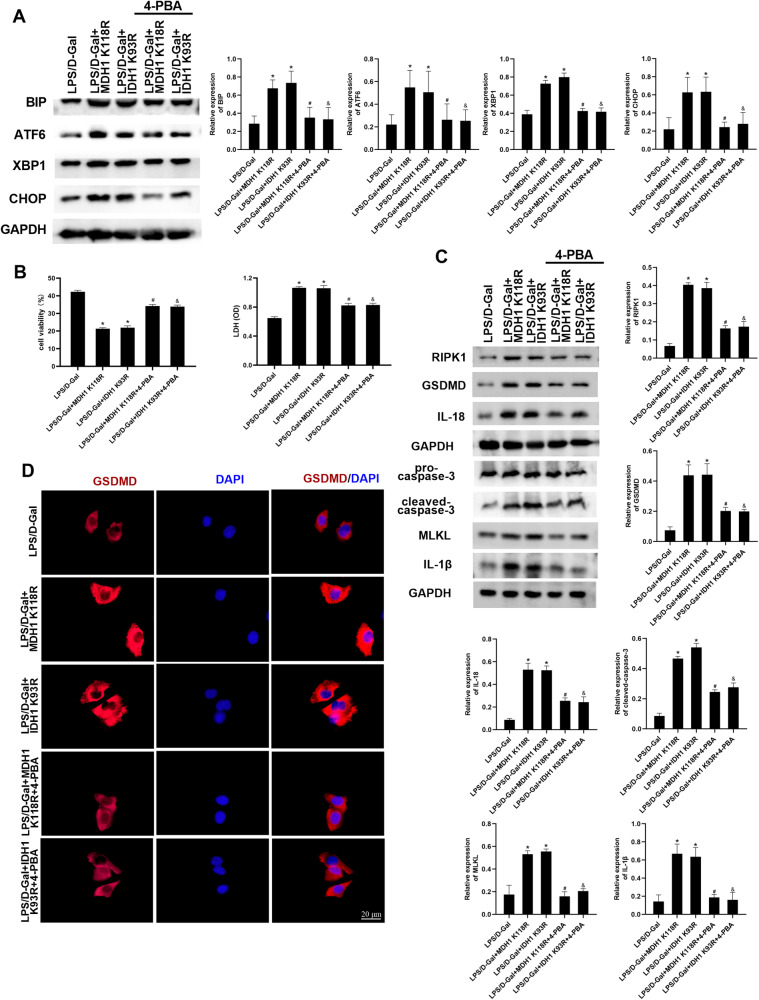
Deacetylated MDH1 and IDH1 deacetylation aggravated PANoptosis during ALF through ER stress signaling. **A** The expression of ER stress-related molecules BIP, ATF6, XBP1, CHOP in AML-12 cells was detected by Western blot. **B** Cell viability and LDH release of AML-12 cells in each group. **C** The expression of PANoptosis-related molecules RIPK1, GSDMD, caspase-3, MLKL, IL-18, and IL-1β in AML-12 cells was detected by Western blot. **D** The expression of GSDMD in AML-12 cells was detected by immunofluorescence. The results are presented as mean ± SD based on three repetitions. * compared with LPS/D-Gal group, *P* < 0.05; # compared with LPS/D-Gal + MDH1K118R group, *P* < 0.05; & compared with LPS/D-Gal + IDH1 K93R group, *P* < 0.05.

In order to determine if deacetylated MDH1 and IDH1 could aggravate PANoptosis in ALF through ER stress signaling, we intervened ER stress inhibitor 4-PBA in cells. As shown in Fig. 7A, 4-PBA significantly decreased the expression of ER stress-related molecules BIP, ATF6, XBP1, and CHOP. Western blot and immunofluorescence results showed that, compared with the LPS/D-Gal + MDH1 K118R and LPS/D-Gal + IDH1 K93R groups, 4-PBA significantly increased cell viability, decreased LDH release, and reduced the expression of PANoptosis-related molecules RIPK1, GSDMD, caspase-3, MLKL, IL-18 and IL-1β (Fig. 7B–D). The results indicated that 4-PBA weakened the impact of deacetylated MDH1 and IDH1 on PANoptosis. These findings suggested that deacetylated MDH1 and IDH1 may aggravate PANoptosis in ALF through ER stress signaling.

## Discussion

PANoptosis is an extensive form of cell death characterized by pyroptosis, apoptosis, and necroptosis, and plays a crucial role in the body’s defense mechanisms. There is an inseparable interaction between necroptosis, apoptosis, and pyroptosis. The role of PANoptosis in infectious diseases has been widely reported, including bacterial, viral, and fungal infections [18, 19]. But its relationship with ALF, MDH1 and IDH1 acetylation remains unclear.

Our results showed that the expression of PANoptosis-related molecules RIPK1, GSDMD, MLKL, caspase-3, IL-18, and IL-1β was increased during ALF, indicating an elevated level of PANoptosis during ALF. The caspase family and GSDMD are important molecules that regulate pyroptosis, apoptosis, and necroptosis. Caspase-3 is a crucial regulatory molecule of apoptosis. The inflammasome binds to caspase-1, and the activated caspase-1 cleaves IL-1β and IL-18 precursors, promoting the release of IL-1β and IL-18, and mediating cell death [8]. Activated caspases can also cleave GSDMD to initiate cell death [9]. RIPK1 is a molecule required for the regulation of PANoptosis and inflammatory responses [20]. Loss of RIPK1 can eliminate pyroptosis and apoptosis induced by Yersinia [5]. RIPK1 can recruit NLRP3 and ASC to form a cell death complex, activate the inflammasome, caspase-8, and GSDMD, leading to cell pyroptosis and apoptosis [4, 21]. RIPK1 is also involved in tumor necrosis factor-α (TNF-α) and interferon-γ (IFN-γ)-induced PANoptosis driven by the FADD/caspase-8 signaling. Therefore, targeting key molecules of PANoptosis may be a new way to treat ALF.

Our results also found that deacetylated MDH1 and IDH1 could enhance the expression of PANoptosis-related molecules such as RIPK1, GSDMD, MLKL, caspase-3, and other molecules in ALF. This suggested that deacetylated MDH1 and IDH1 could aggravate PANoptosis during ALF. MDH1 and IDH1 are two important metabolic enzymes involved in energy metabolism. Studies have reported that the acetylation of MDH1 and IDH1 can affect their activities and function in diseases [22, 23]. In our previous studies, we found that the deacetylation of MDH1 K118 and IDH1 K93 reduced the activity and function of MDH1 and IDH1, respectively [15, 16]. The dysfunction of MDH1 and IDH1 can disrupt energy metabolism. Cell death is closely related to energy metabolism and mitochondria [24]. Necroptosis is characterized by mitochondrial swelling, loss of mitochondrial membrane potential, impaired oxidative phosphorylation, and ATP production [24]. Anoikis is a unique form of apoptosis. Cells undergo drastic metabolic changes in Anoikis, characterized by reduced glucose uptake, glycolytic flux, mitochondrial respiration, and pentose phosphate pathway, as well as decreased ATP and NADPH production [25]. The Bcl-2 family, which regulates apoptosis, is influenced by various metabolic stresses. Glucose metabolism can also affect the activation of the pro-apoptotic molecule Bax [26, 27]. Therefore, cell death is inseparable from the regulation of energy metabolism. In addition to their basic role in energy metabolism, mitochondria also play a crucial role in regulating various forms of cell death, with the most extensively studied being their involvement in apoptosis. In the exogenous apoptotic pathway, ligands bind to receptors, resulting in the formation of a death complex that activates caspase-8. Caspase-8 can activate downstream caspase cascades, including caspase-3, which targets hundreds of substrates, leading to apoptosis. Mitochondria play a role in amplifying the cycle of exogenous apoptosis [28]. Mitochondria play a crucial role in endogenous apoptosis. The endogenous apoptotic pathway is also known as the mitochondrial apoptotic pathway. Signals such as DNA damage, oxidative stress, or endoplasmic reticulum stress act on mitochondria, stimulate the permeabilization of the mitochondrial outer membrane, and lead to the release of pro-apoptotic factors from mitochondria [28–30].

Our results also suggest that the mechanism by which deacetylated MDH1 and IDH1 promote PANoptosis during ALF may be through the ER stress signaling. Physiological and pathological stimuli, such as nutritional deprivation, oxidative stress, hypoxia, and genetic mutations, can disrupt ER homeostasis and lead to ER stress [31]. In response to ER stress, cells activate the unfolded protein response (UPR) to adapt to ER stress or undergo cell death. During mild to moderate ER stress, the UPR is activated to eliminate unfolded or misfolded proteins and restore ER homeostasis. However, under severe or persistent ER stress, the UPR can be overactivated, leading to the initiation of cell death [32]. When unfolded or misfolded proteins accumulate inside the cell, they bind to BIP, competitively dissociating BIP from the UPR sensor, causing BIP to activate [33]. After BIP dissociation, IRE1α was autophosphorylated [34]. IRE1α autophosphorylation activates XBP1 and enhances the transcriptional expression of CHOP, thereby promoting cell death. In addition, under ER stress, ATF6 is transported to the Golgi apparatus in a vesicular manner. There, it is cleaved and activated, leading to the transcriptional expression of ER stress genes, such as CHOP. CHOP is considered one of the key factors of ER stress and is widely involved in the pathogenesis of liver diseases [35, 36]. Therefore, excessive ER stress can lead to the activation of cell death. Our results showed that deacetylated MDH1 and IDH1 could impact ER stress signaling, indicating a strong connection between metabolism, mitochondria, and ER stress. MDH1 and IDH1 are two important metabolic enzymes involved in energy metabolism. The dysfunction of MDH1 and IDH1 can disrupt energy metabolism. Studies have shown that the activation level of the UPR pathway changes with metabolic status in various mammalian tissues [37, 38]. Both carbohydrate metabolism and lipid metabolism can influence ER stress. Glucose metabolism is related to protein folding and translocation. Glucose metabolism imbalance can activate ER stress and UPR [39]. Excessive lipid exposure can also lead to ER stress, which, in turn, disrupts lipid metabolism and ultimately compromises the protective mechanisms of the ER [40, 41]. Therefore, the influence of energy metabolism imbalance caused by MDH1 and IDH1 on ER stress may be multifaceted.

In summary, this study explored the effect and possible mechanism of deacetylated MDH1 and IDH1 on PANoptosis in ALF. The results indicated that deacetylated MDH1 and IDH1 could aggravate PANoptosis during ALF, and the mechanism may involve the ER stress signaling. Targeting MDH1 and IDH1 acetylation modifications, as well as PANoptosis-related molecules, may represent a novel strategy for treating ALF.

## Materials and methods

### Reagents

Foetal bovine serum (FBS) and DMEM/F12 medium were obtained from Gibco (USA). D-Gal and LPS were purchased from Sigma-Aldrich (USA). ACY1215 (#HY-16026) and 4-PBA (#HY-A0281) were purchased from MCE (USA). IDH1 (#12332-1-AP), MDH1 (#15904-1-AP), RIPK1 (#17519-1-AP), GSDMD (#20770-1-AP), IL-18 (#10663-1-AP), MLKL (#21066-1-AP), ATF6 (#24169-1-AP), BIP (#11587-1-AP), XBP1 (#24868-1-AP), CHOP (#15204-1-AP), and GAPDH (#60004-1-Ig) specific antibodies were obtained from Proteintech (China). Acetylated lysine (Ac-Lys) antibody (#9441) was purchased from Cell Signaling Technology (USA). The IL-1β antibody (#ab254360) was purchased from Abcam (USA). The caspase-3 antibody (#sc-56053) was purchased from Santa Cruz Biotechnology (USA).

### Cell culture and treatment

The mouse liver cell line AML-12 was cultured in DMEM/F12 supplemented with 10% FBS, sourced from Wuhan Pinuofei Biological. The source of cell line was recently authenticated and determined to be free of mycoplasma. LPS (100 ng/mL) combined with D-Gal (44 μg/mL) [42] was used to stimulate cells, except for those in the control group. ACY1215 (2.5 μM) was added 2 h before LPS/D-Gal [43]. The cells were harvested 24 h after LPS/D-Gal administration.

### Plasmid transfection

MDH1 K118R and IDH1 K93R mutants were constructed by Genomeditech (China). Cells were seeded into 6-well plates. Serum-free medium and plasmids were added to a centrifuge tube and mixed. Seru-free medium and Lipofectamine 2000 were added to another centrifuge tube. After 5 min at room temperature, the two tubes were mixed and incubated for 20 min at room temperature. The transfection mixture was added to the cell culture plate and mixed. Cells were intervened after 24–72 h transfection. LPS/D-Gal and ACY1215 were used to stimulate the cells after plasmid transfection, as described above.

### Immunofluorescence

After treatment, the cells were fixed with 4% paraformaldehyde for 30 min, permeabilized with 0.2% Triton for 15 min, blocked with 5% bovine serum albumin (BSA) for 30 min, and incubated with primary antibodies (1:100) overnight at 4°C and secondary antibodies (Servicebio, China) for 1 h at room temperature. The results were observed under a fluorescence microscope (Olympus, Japan). After dewaxing and antigen retrieval, tissue slices were blocked with BSA for 1 h, incubated with primary antibodies (1:100) overnight at 4 °C, and then with secondary antibodies for 1 h at room temperature. The results were observed using a fluorescence microscope (Olympus, Japan).

### PI staining

After being treated, the cells were washed with phosphate-buffered saline (PBS) and then incubated with a Calcein AM/PI dye solution at 37 °C for 30 min to 1 h, avoiding light. After incubation, the results were observed under a fluorescence microscope (Olympus, Japan).

### Cell viability

The CCK-8 kit was used to assess cell viability. After being treated, the cells were washed with PBS and then incubated with CCK-8 testing solution at 37 °C for 1 to 2 h. Following the incubation, the results were measured at 450 nm using a microplate reader (Perkin Elmer, USA).

### LDH release

After being treated, the cell supernatant was collected and incubated with LDH testing solution at room temperature for 30 min, avoiding light, according to the instructions. After incubation, the results were detected at 490 nm using a microplate reader (Perkin Elmer, USA).

### Western blotting and immunoprecipitation

The samples were lysed, incubated with 5 μg of primary antibodies or IgG, followed by incubation with 30 μl of protein A + G agarose. Subsequently, they were washed with immunoprecipitation buffer, resuspended in 40 μl of 1.5× loading buffer, boiled, and centrifuged. The supernatants were collected for Western blot analysis. Sodium dodecyl sulfate polyacrylamide gel electropheresis (SDS-PAGE) was used to separate the proteins. After electrophoresis, the proteins were transferred to a polyvinylidene fluoride (PVDF) membrane (Millipore, USA). Subsequently, the membrane was cut and incubated with specific primary and secondary antibodies. GAPDH was used as a loading control. The results were detected by a chemiluminescence apparatus (Bio-Rad, USA). The Image J software was used to analyze the quantification of blots.

### Animal groups

Seventy-two male C57BL/6 mice (6-8 weeks old, 20-25 g) were obtained from the Experimental Animal Center of Wuhan University. MDH1 K118R and IDH1 K93R adeno-associated viruses were constructed for transfection in mice (AAV8, Genomeditech, China). The mice were acclimated for 5 days and then randomly and blindingly divided into twelve groups: EV group, WT group, MDH1 K118R group, IDH1 K93R group, EV + LPS/D-Gal group, WT + LPS/D-Gal group, LPS/D-Gal + MDH1 K118R group, LPS/D-Gal + IDH1 K93R group, EV + LPS/D-Gal + ACY1215 group, WT + LPS/D-Gal + ACY1215 group, LPS/D-Gal + ACY1215 + MDH1 K118R group, and LPS/D-Gal + ACY1215 + IDH1 K93R group. Except for the EV, WT, MDH1 K118R, and IDH1 K93R groups, the remaining mice received intraperitoneal injections of LPS (100 µg/kg) and D-Gal (400 mg/kg) [43]. Adeno-associated viruses were administered via tail vein injection at a dose of 1E11 vg 4 weeks before LPS/D-Gal injection. The ACY1215 (25 mg/kg) was administered via intraperitoneal injection 2 h before the LPS/D-Gal injection [43]. At 24 h after the LPS/D-Gal injection, the mice were sacrificed. The livers and serum were collected for experiments. The animal experiments were conducted in accordance with ARRIVE guidelines and other relevant regulations. Approval was granted by the Institutional Animal Care and Use Committee of Renmin Hospital of Wuhan University.

### Hematoxylin-eosin (HE) staining and detection of ALT, AST, and TBIL

Fresh tissues were fixed with 4% paraformaldehyde for 24 h at room temperature, dehydrated through a serial alcohol gradient, embedded in paraffin, processed for sectioning. Before staining, sections were dewaxed in xylene, rehydrated through decreasing concentrations of ethanol, and washed in PBS. And then stained with hematoxylin and eosin. After staining, sections were dehydrated through increasing concentrations of ethanol and xylene. The results were analyzed under a microscope (Olympus, Japan). ALT, AST, and TBIL levels in mouse serum were tested using a fully automatic biochemical analyzer (ADVIA 2400, Siemens AG).

### Statistical analysis

All data were analyzed using SPSS 25.0. GraphPad Prism 8.0 software was used to generate the figures. The results are presented as mean ± standard deviation. Analysis of variance (ANOVA) followed by a post-test was used to analyze the differences between groups. *P* < 0.05 was considered statistically significant.

### Supplementary information

Original Western Blots

## Data Availability

The datasets used and analyzed during the current study are available from the corresponding author.
