# Supplementary material for: Deacetylated MDH1 and IDH1 aggravates PANoptosis in acute liver failure through endoplasmic reticulum stress signaling
Source: Cell Death Discov. 2024 Jun 8;10:275. doi: 10.1038/s41420-024-02054-8 (PMC11162427; doi:10.1038/s41420-024-02054-8)
Supplement: Supplementary file 1 — Original Western Blots [file 41420_2024_2054_MOESM1_ESM.pdf]

Uncropped Western blots

Figure 1D

Repeat 1

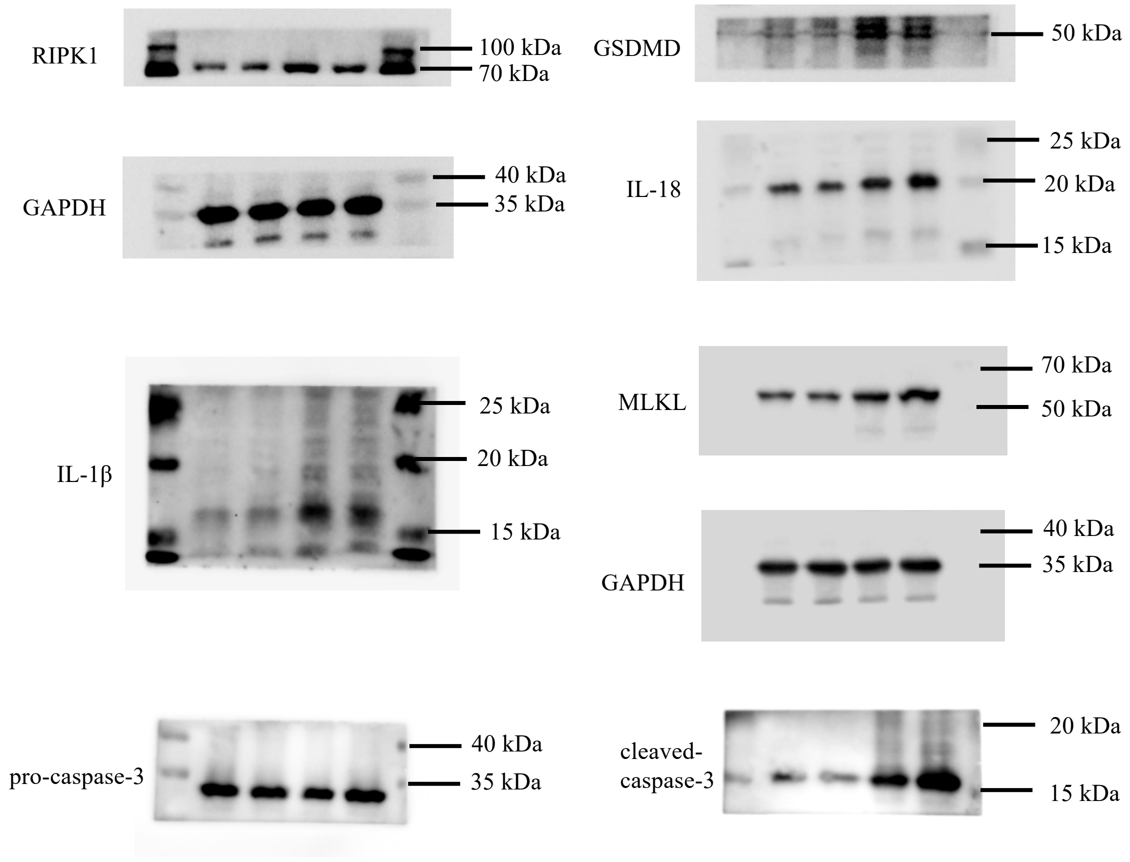

Repeat 2

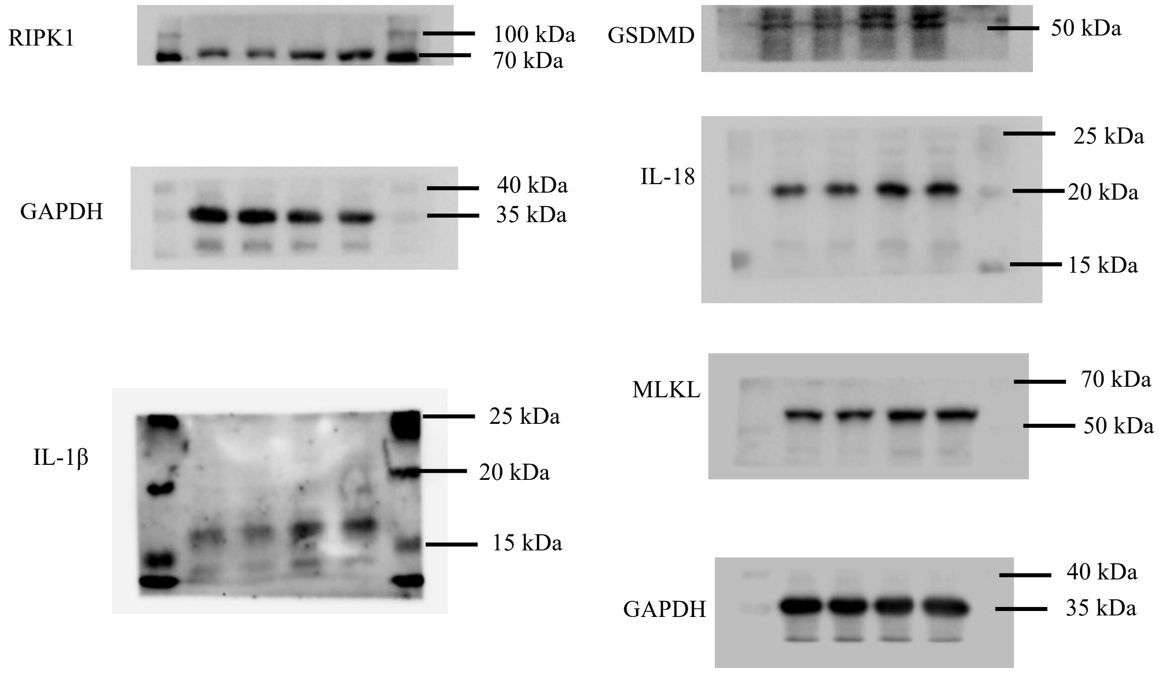

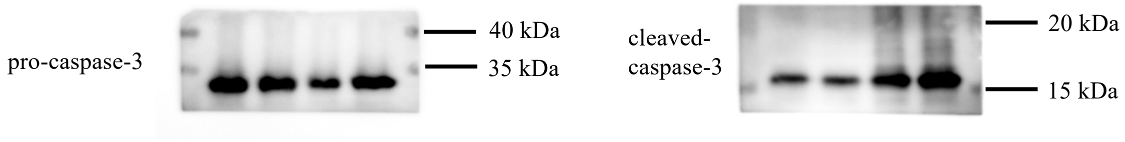

Repeat 3

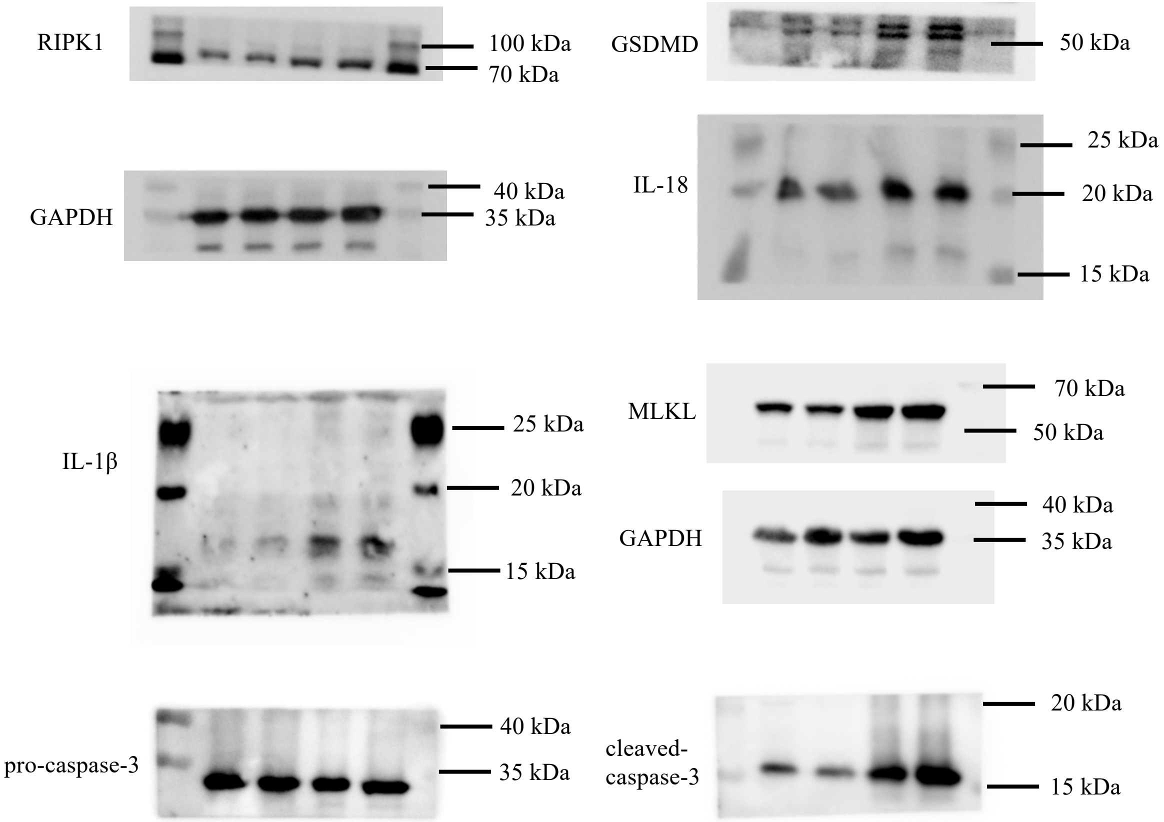

Figure 2A

Repeat 1

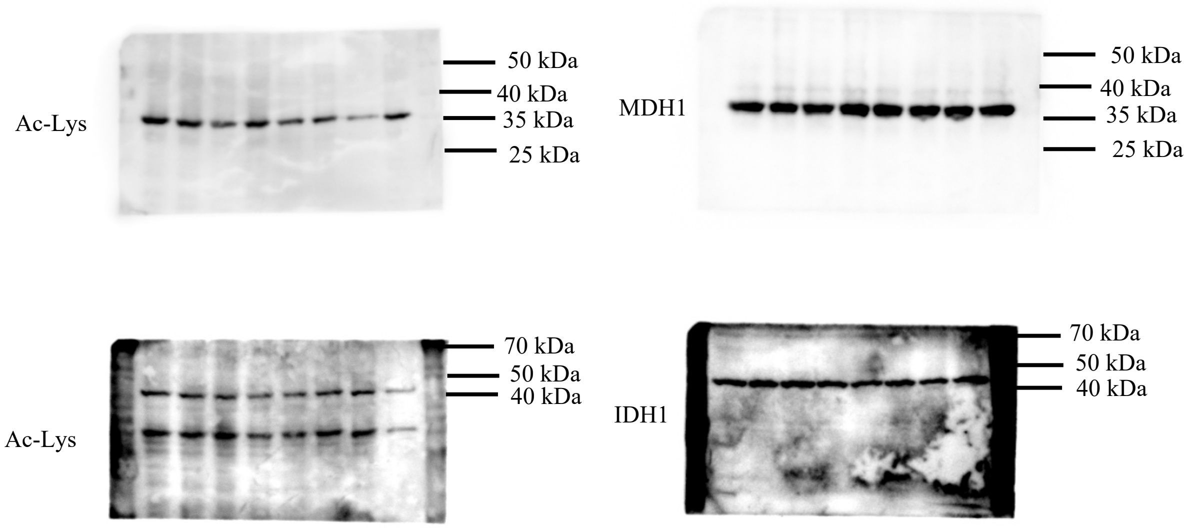

Repeat 2

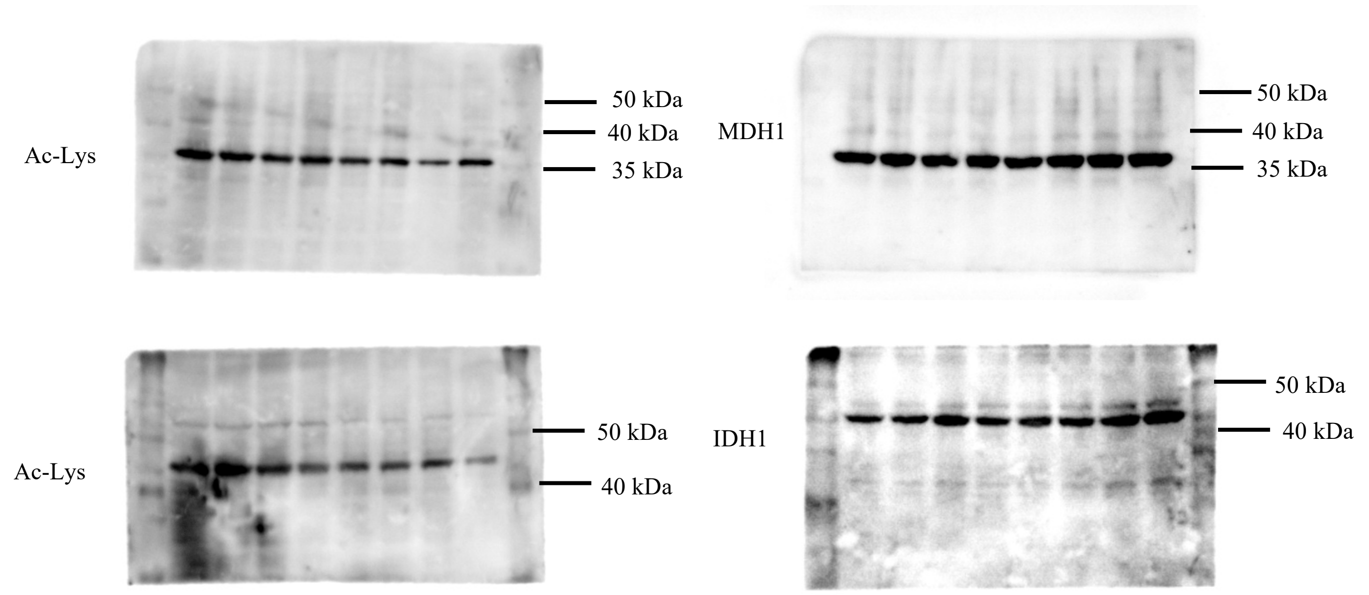

Repeat 3

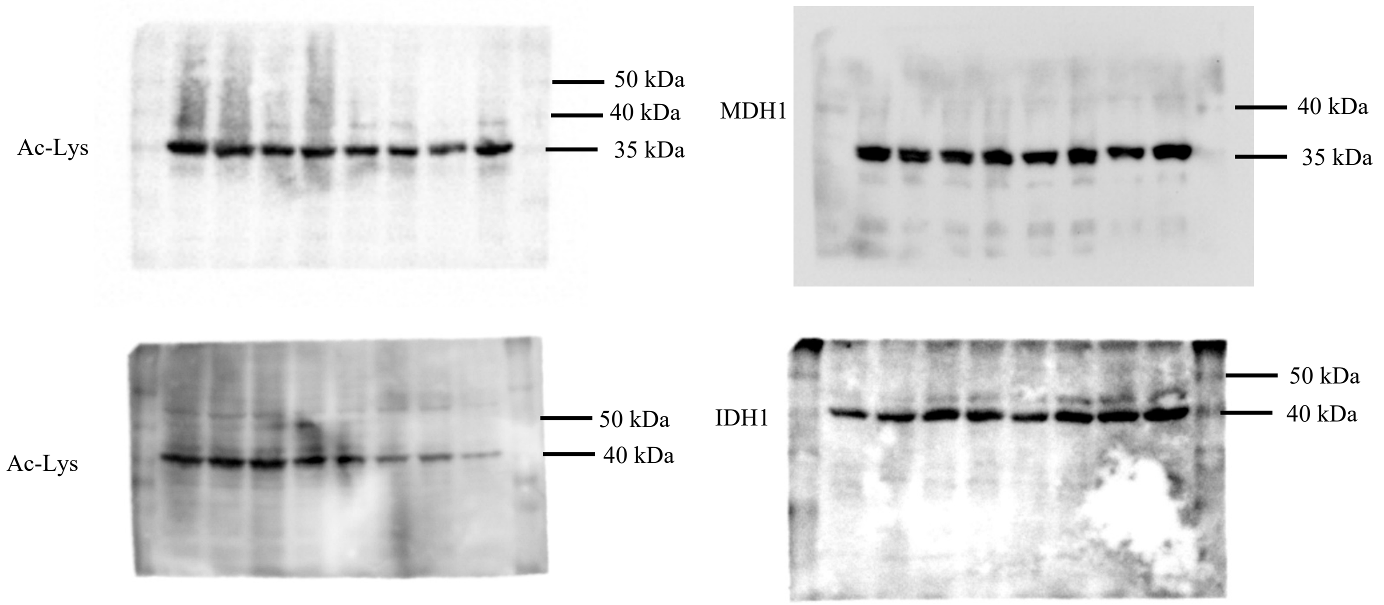

Figure 2D

Repeat 1

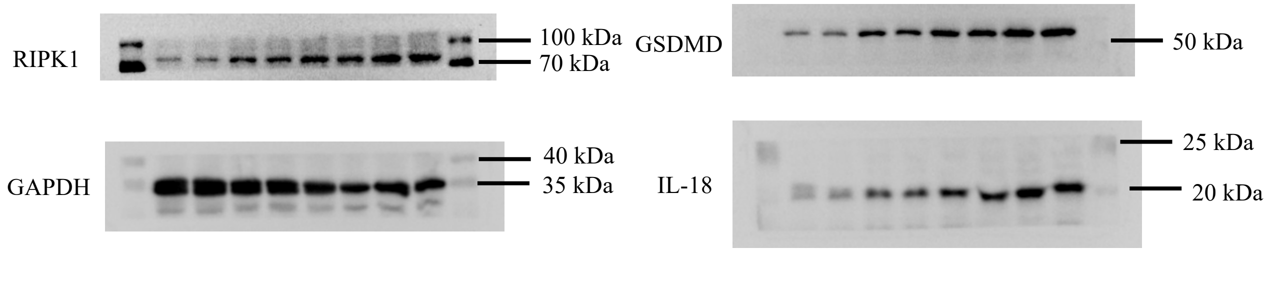

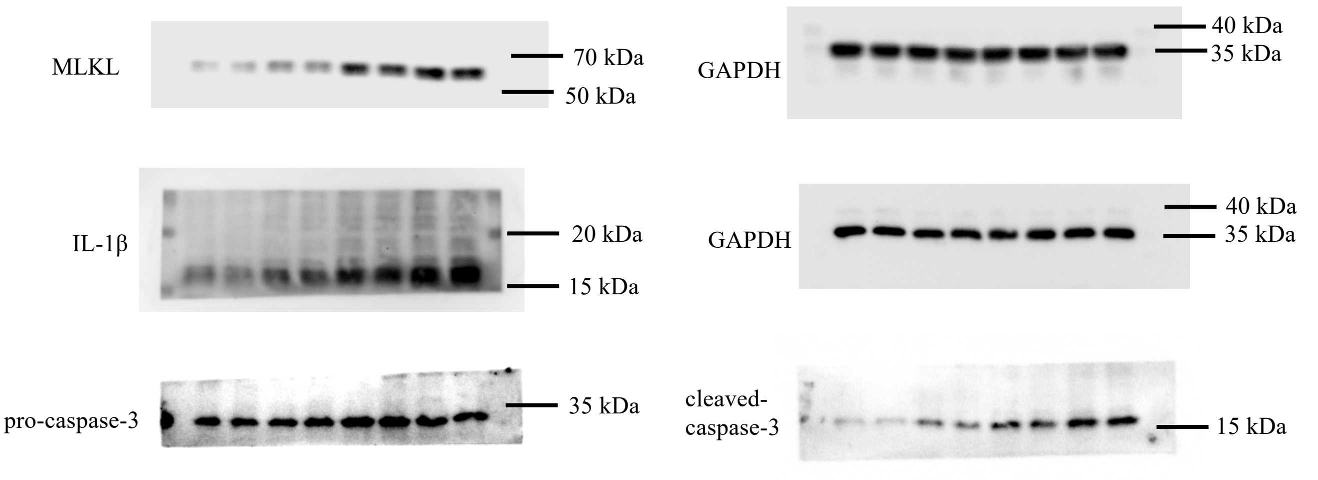

Repeat 2

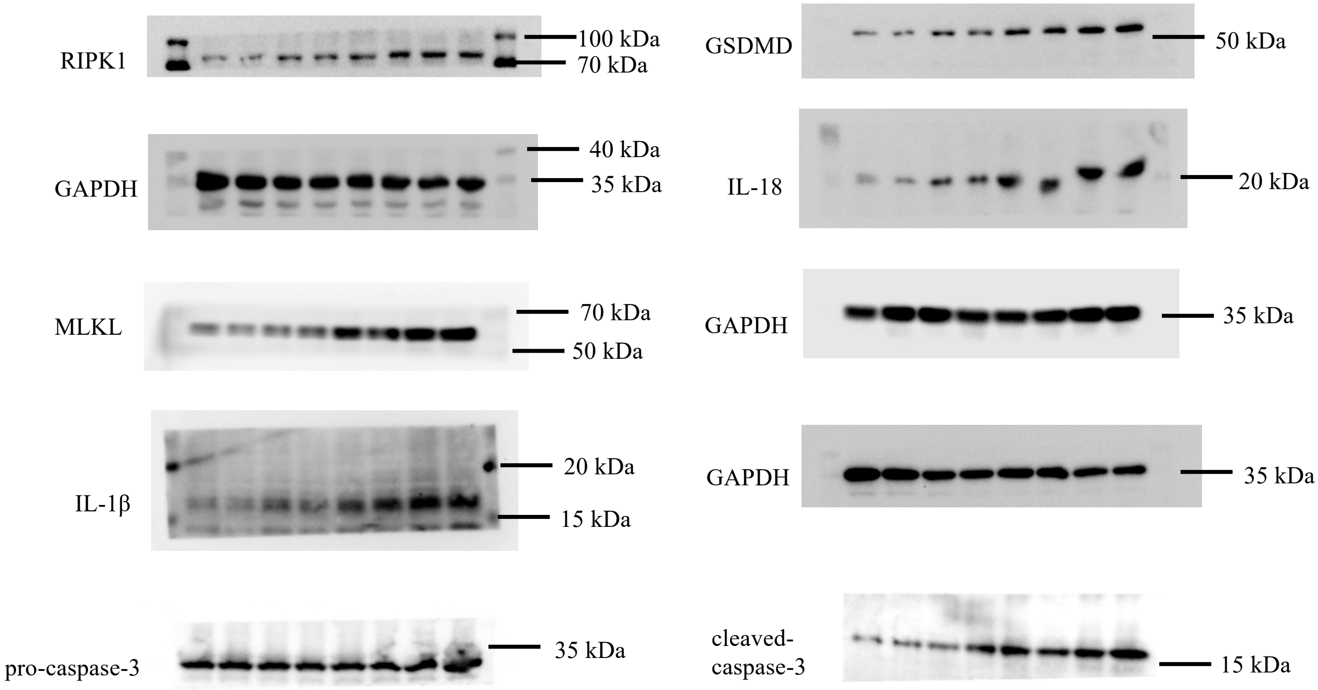

Repeat 3

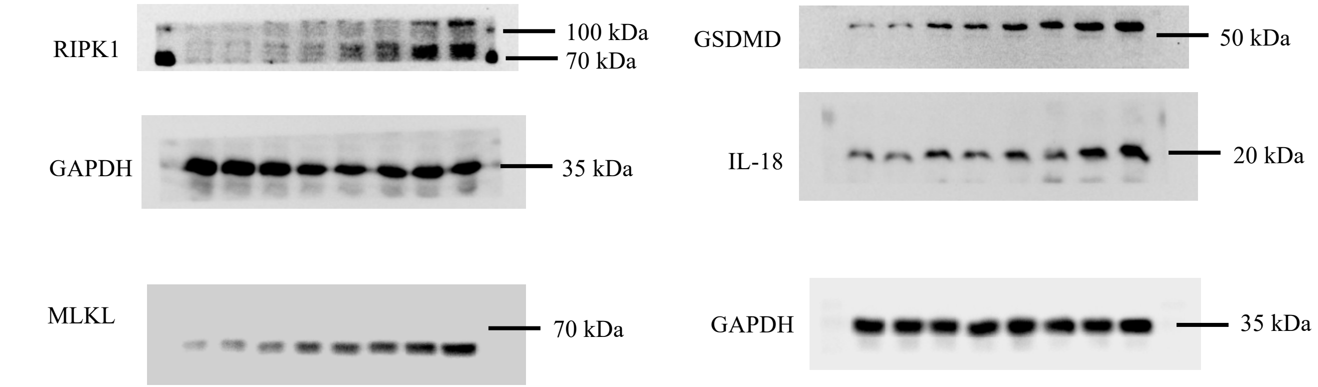

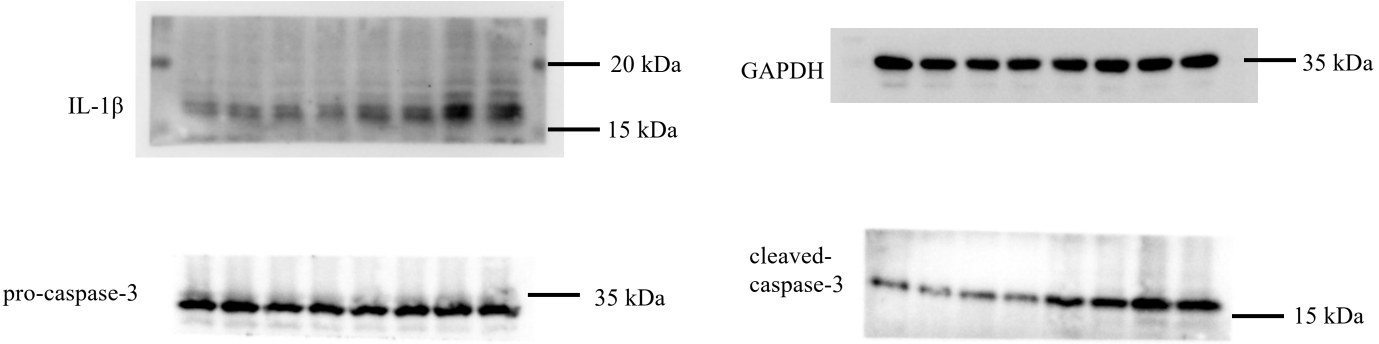

Figure 3A

Repeat 1

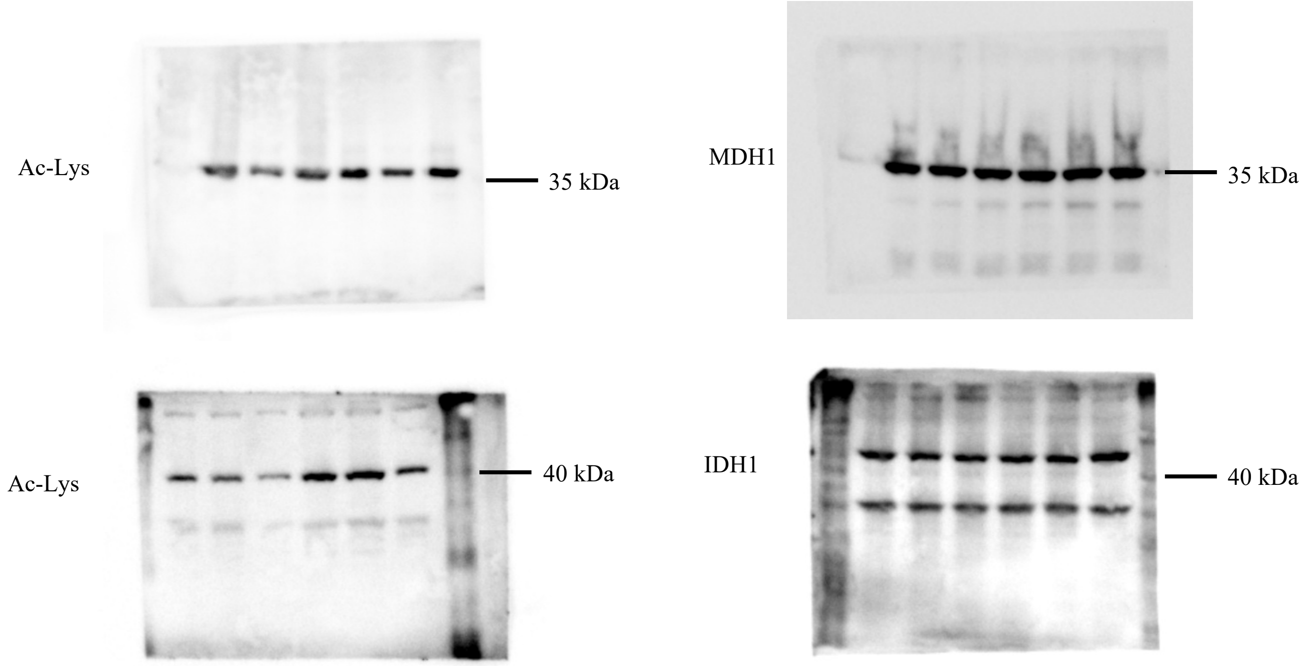

Repeat 2

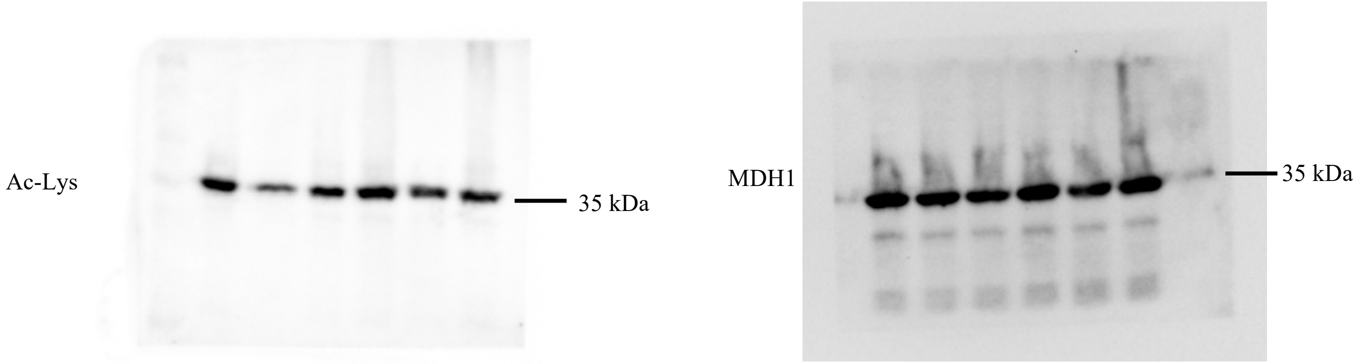

Ac-Lys

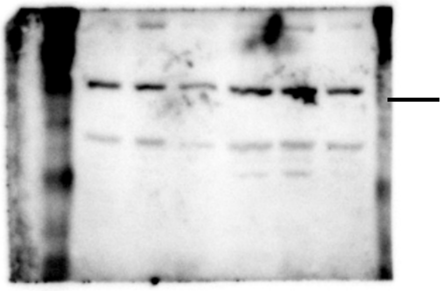

40 kDa

IDH1

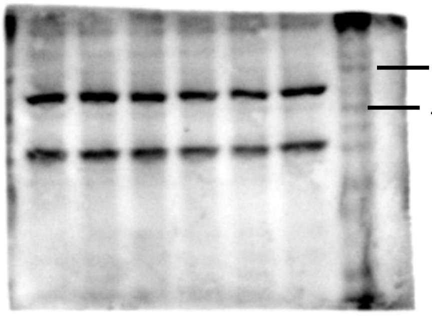

50 kDa
40 kDa

Repeat 3

Ac-Lys

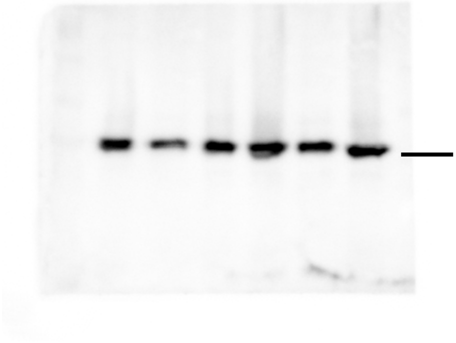

35 kDa

MDH1

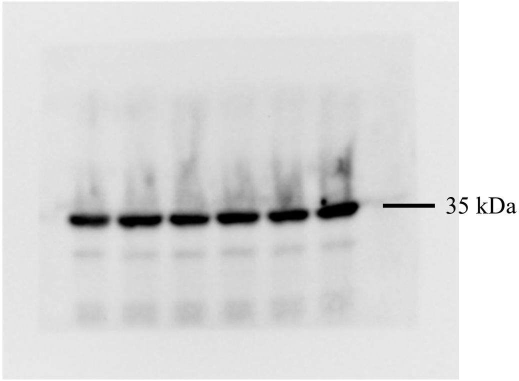

35 kDa

Ac-Lys

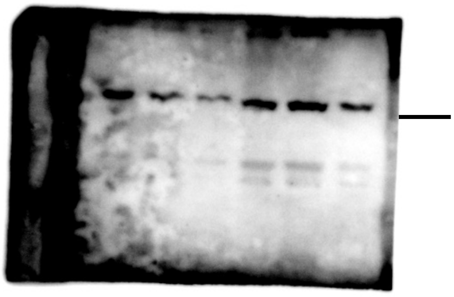

40 kDa

IDH1

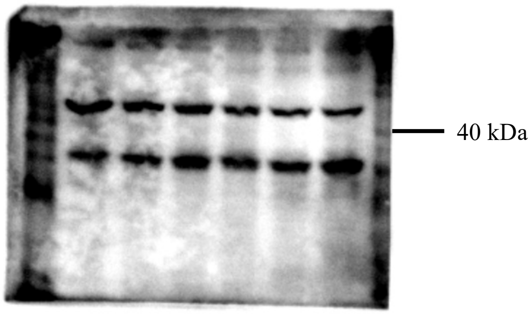

40 kDa

Figure 3D

Repeat 1

RIPK1

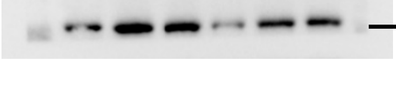

70 kDa

GSDMD

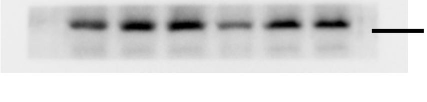

50 kDa

GAPDH

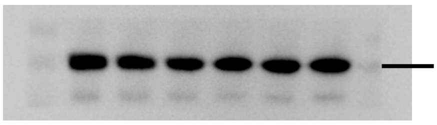

35 kDa

IL-18

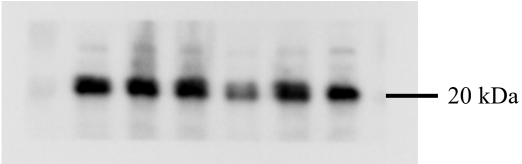

20 kDa

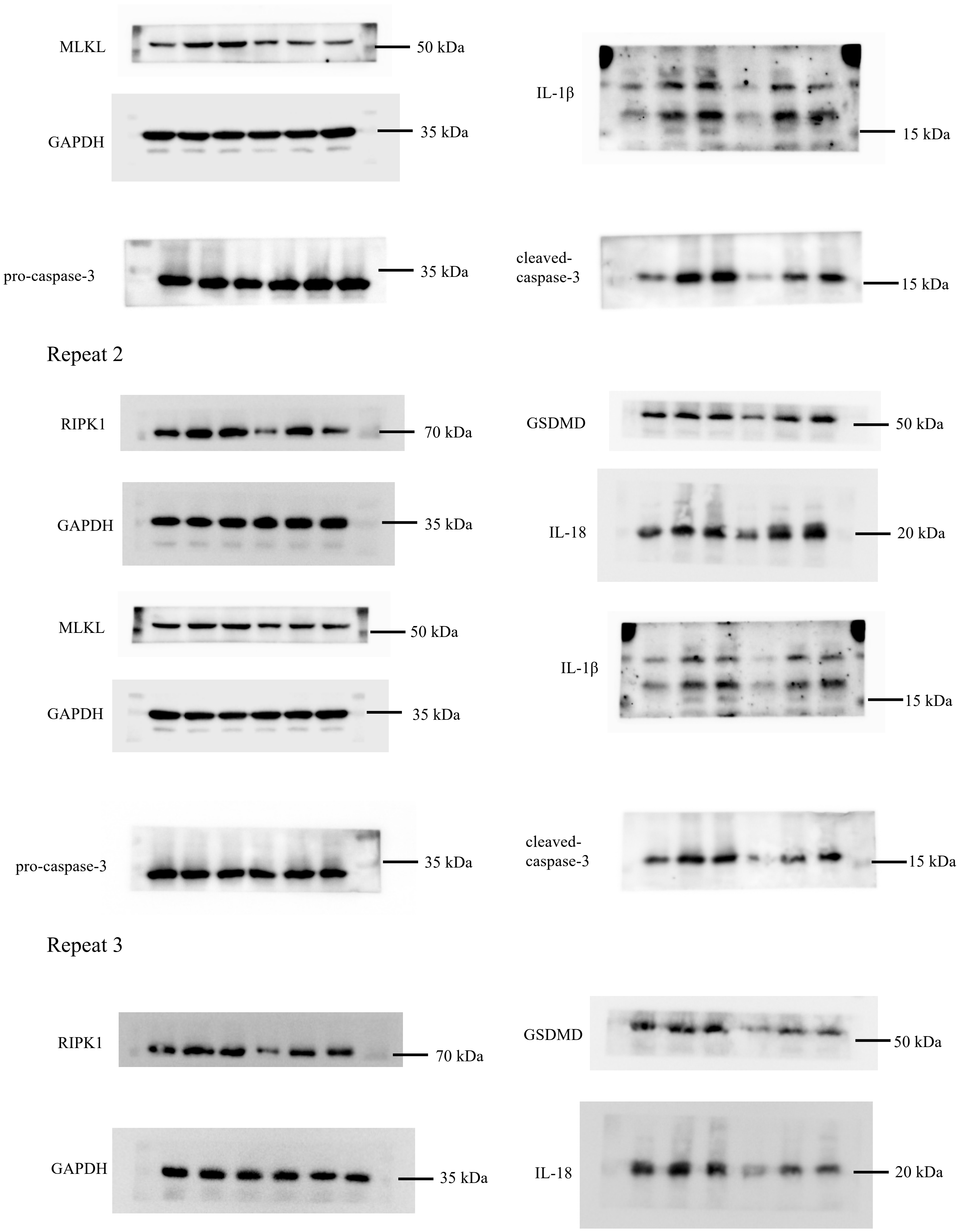
MLKL
50 kDa
GAPDH
35 kDa
IL-1β
15 kDa
pro-caspase-3
35 kDa
cleaved-
caspase-3
15 kDa
Repeat 2
RIPK1
70 kDa
GSDMD
50 kDa
GAPDH
35 kDa
IL-18
20 kDa
MLKL
50 kDa
IL-1β
GAPDH
35 kDa
15 kDa
pro-caspase-3
35 kDa
cleaved-
caspase-3
15 kDa
Repeat 3
RIPK1
70 kDa
GSDMD
50 kDa
GAPDH
35 kDa
IL-18
20 kDa

MLKL
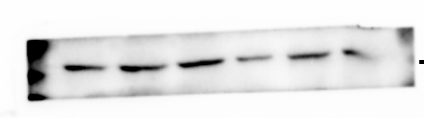
50 kDa

GAPDH
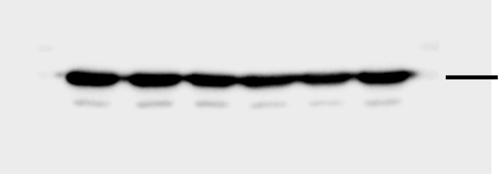
35 kDa

IL-1β
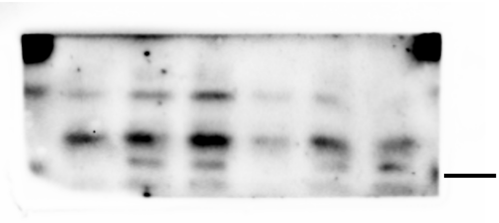
15 kDa

pro-caspase-3
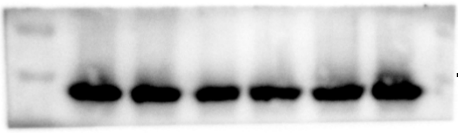
35 kDa

cleaved-caspase-3
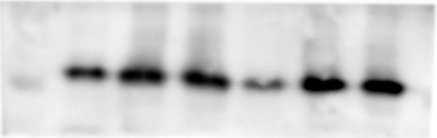
15 kDa

Figure 4B

Repeat 1

Ac-Lys
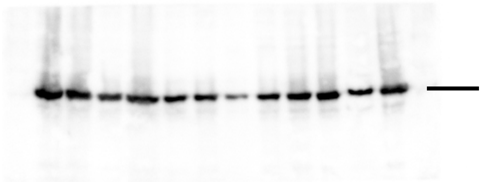
35 kDa

MDH1
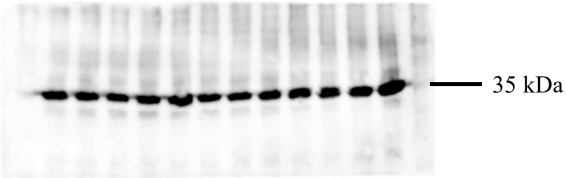
35 kDa

Ac-Lys
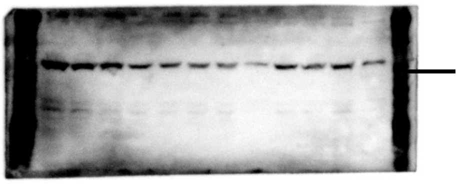
40 kDa

IDH1
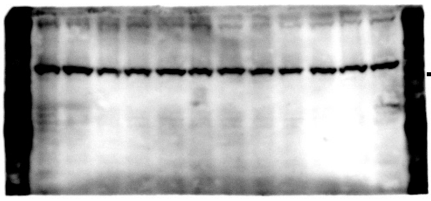
40 kDa

Repeat 2

Ac-Lys
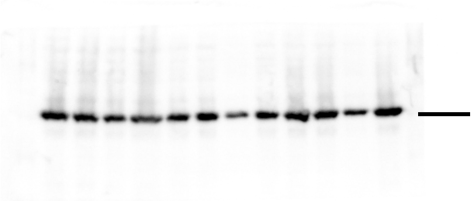
35 kDa

MDH1
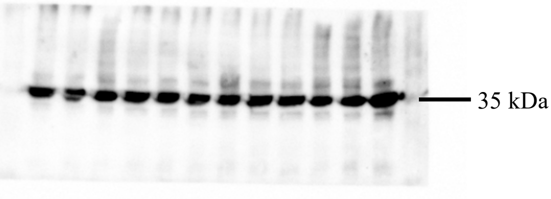
35 kDa

Ac-Lys
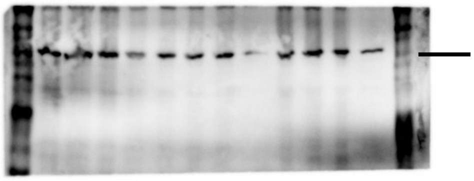
40 kDa

IDH1
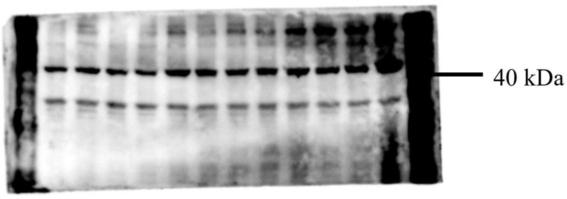
40 kDa

Repeat 3

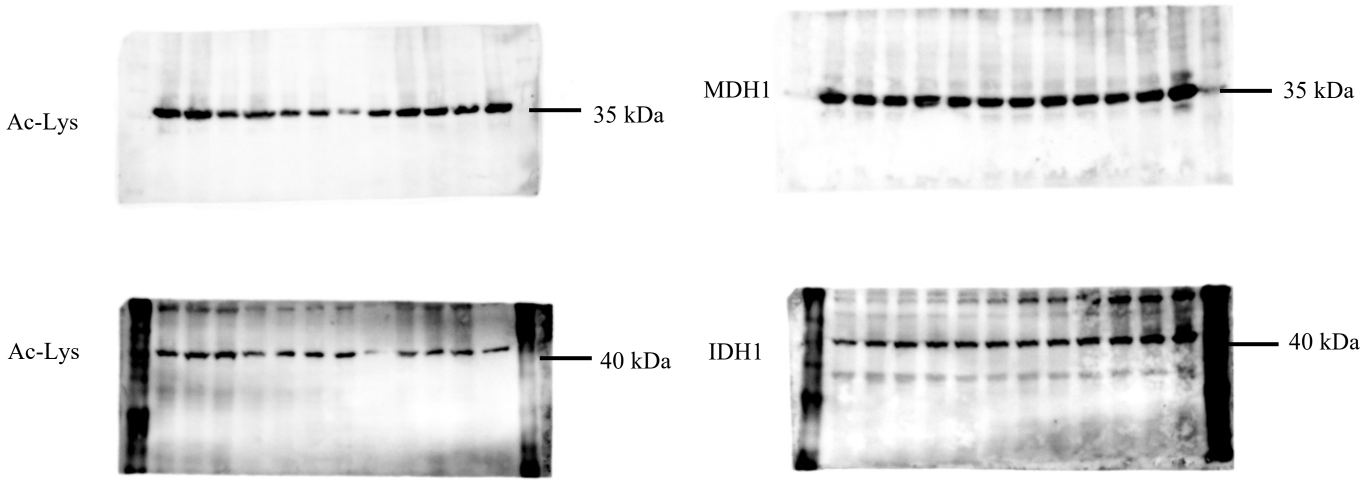

Figure 5A

Repeat 1

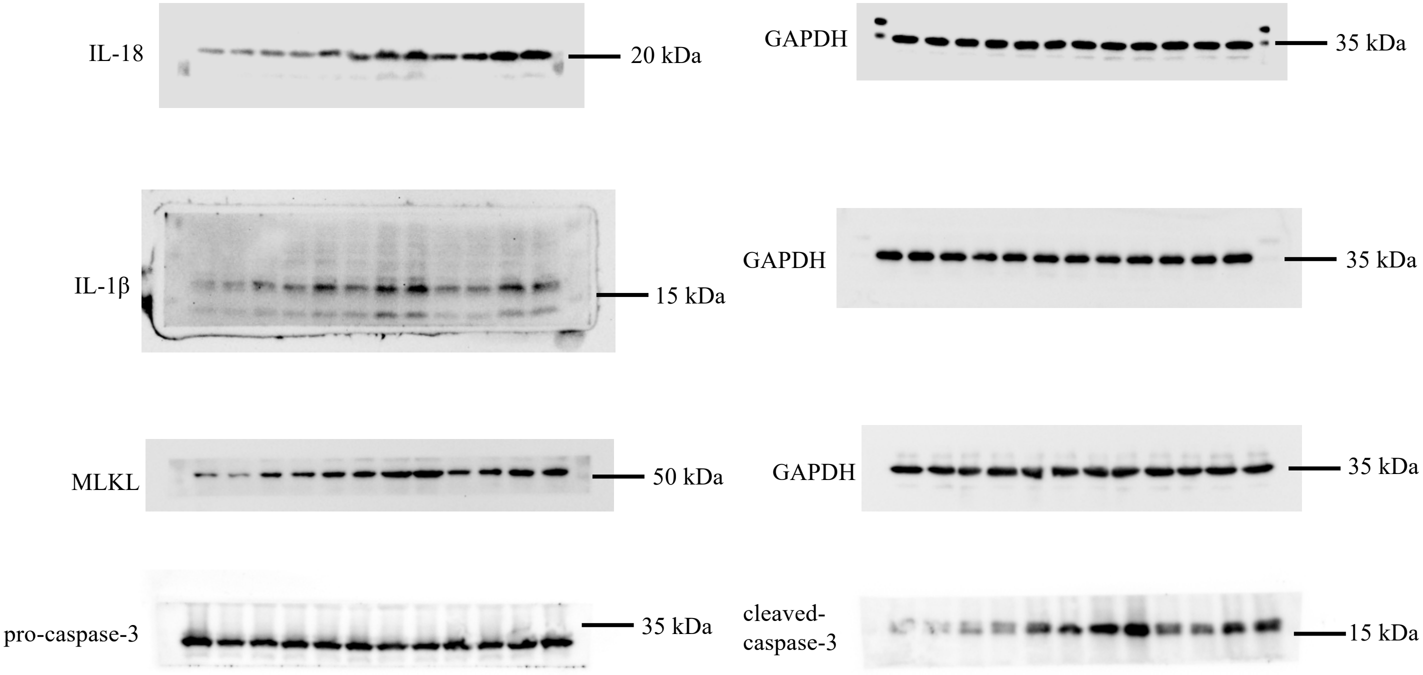

Repeat 2

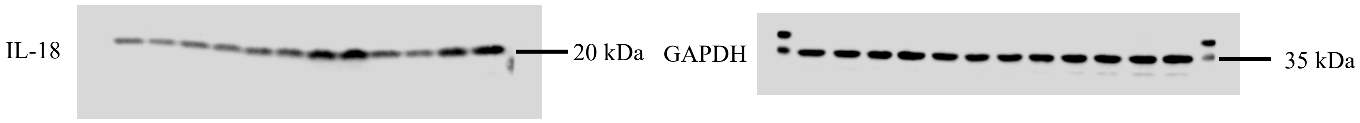

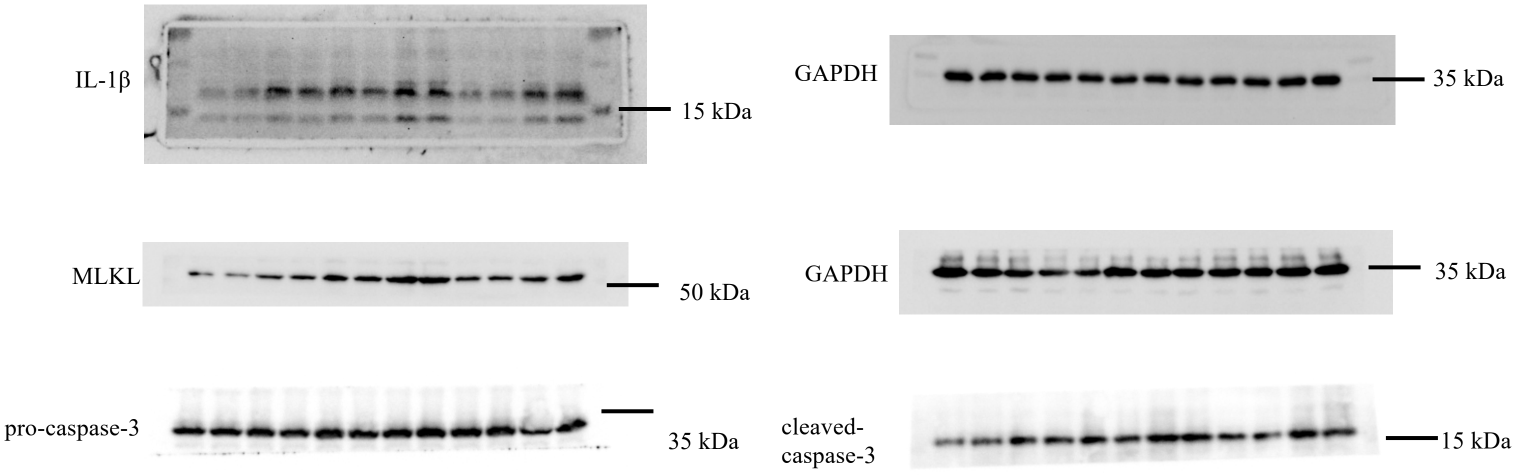

Repeat 3

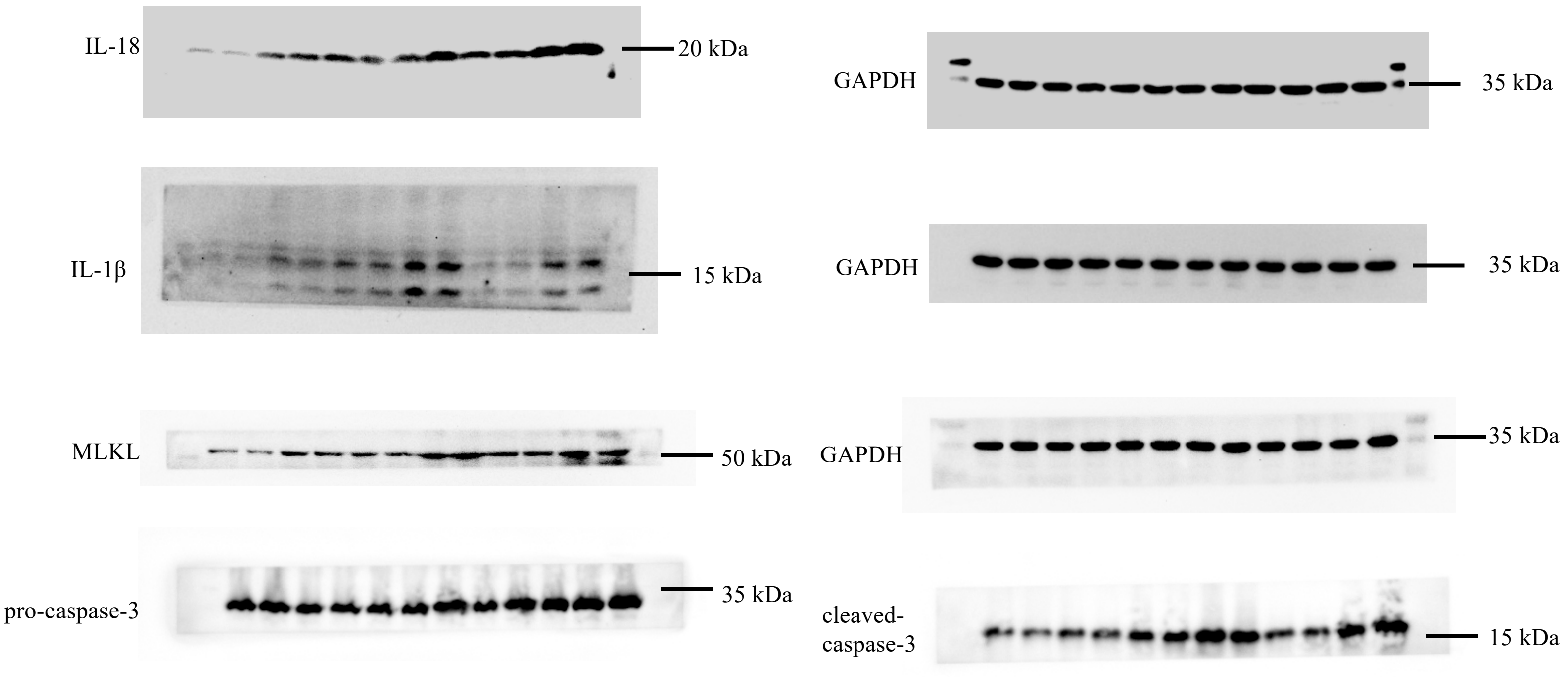

Figure 6A

Repeat 1

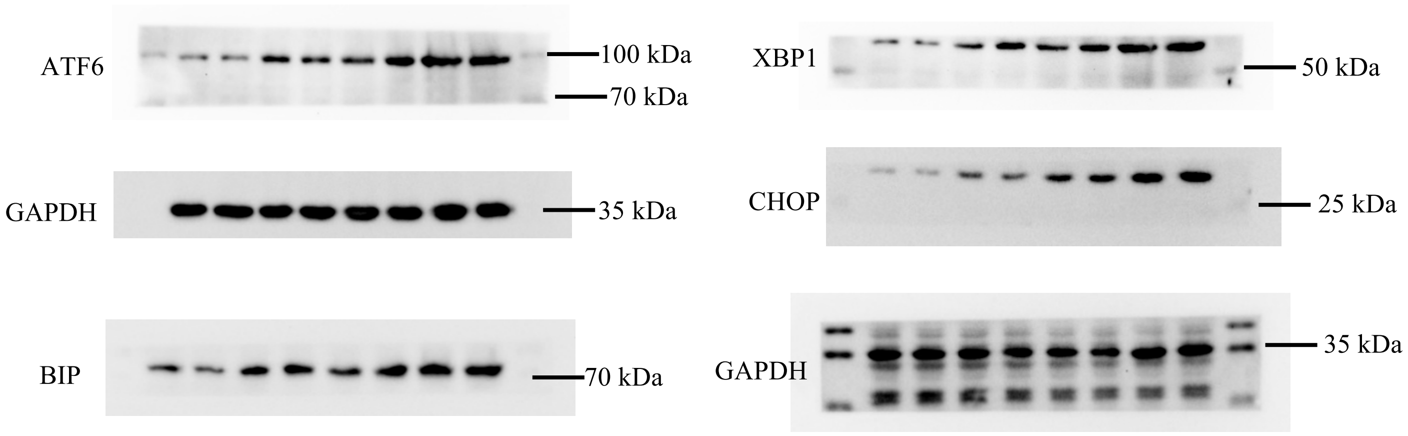

Repeat 2

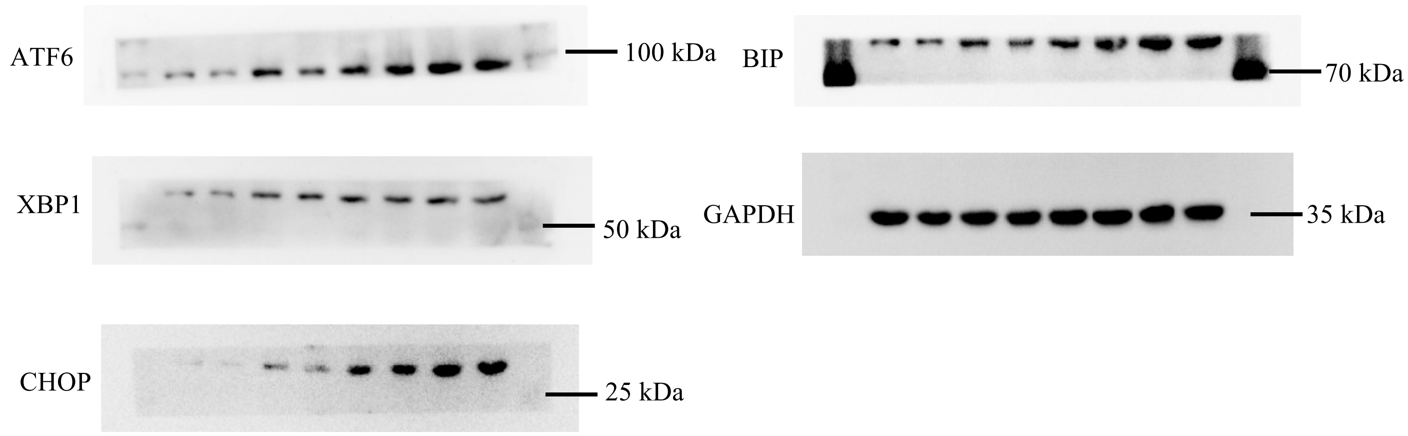

Repeat 3

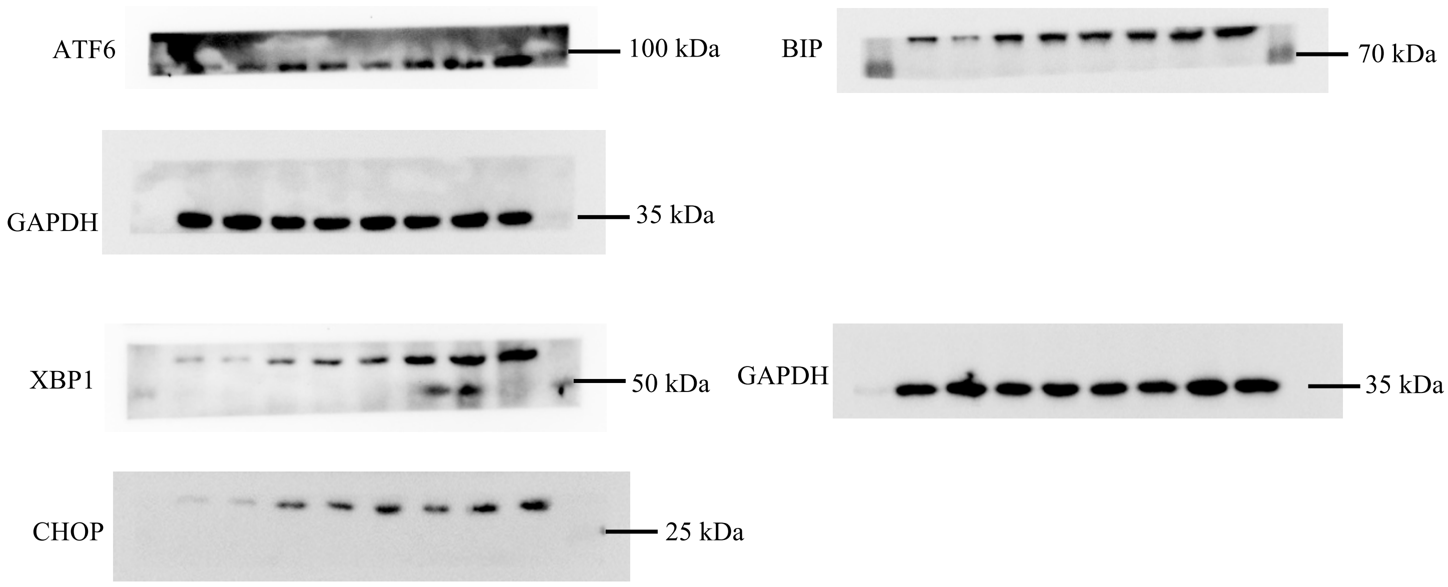

Figure 6B

Repeat 1

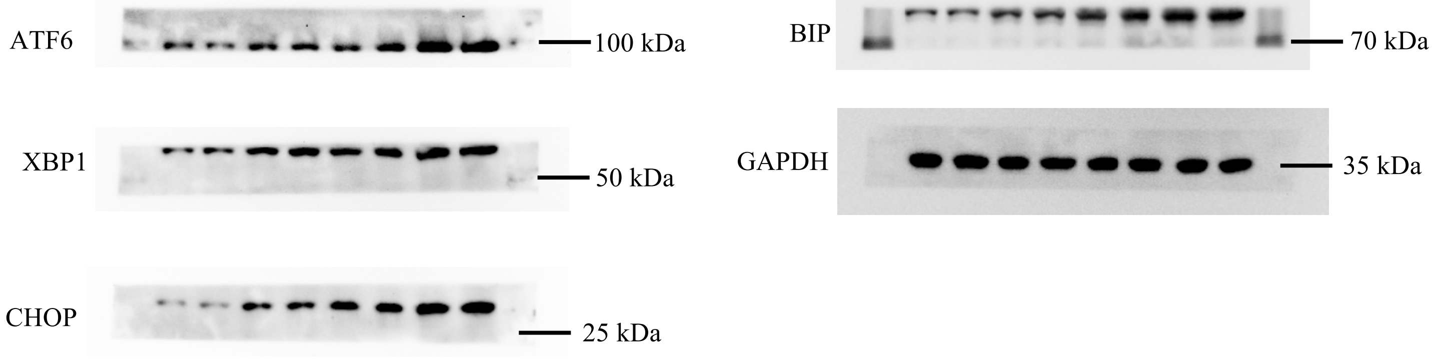

Repeat 2

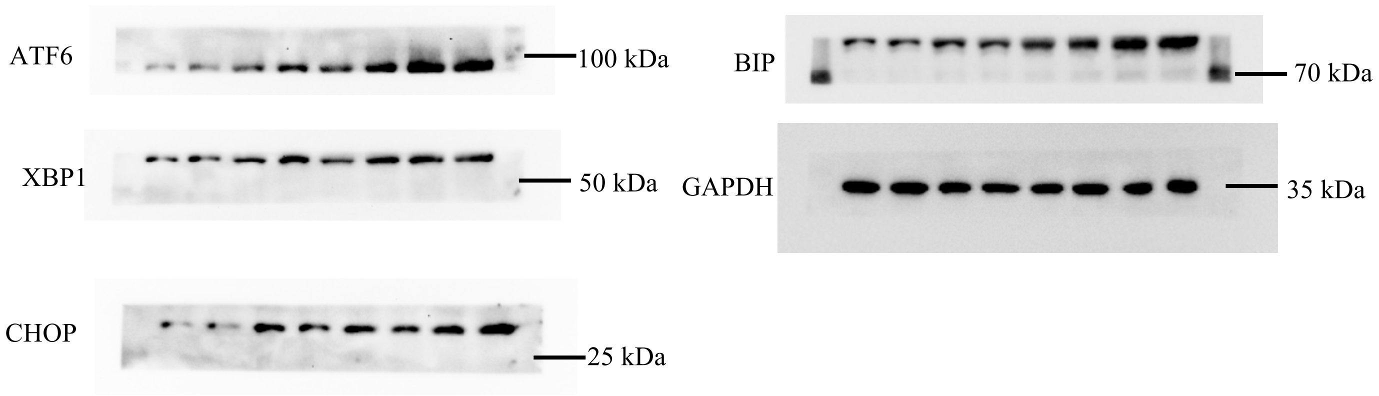

Repeat 3

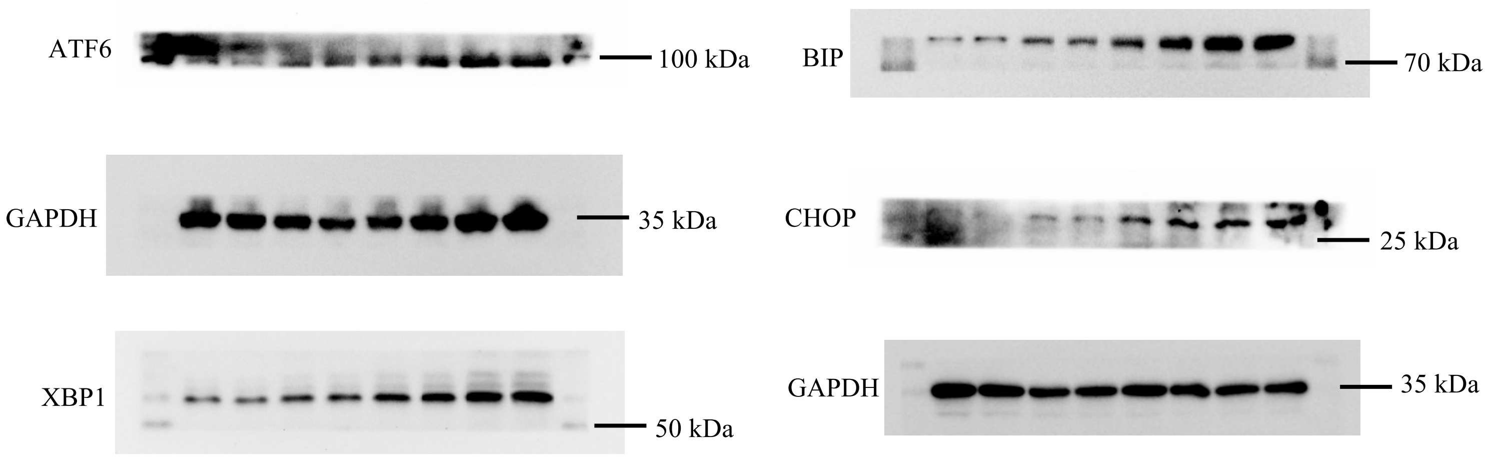

Figure 7A

Repeat 1

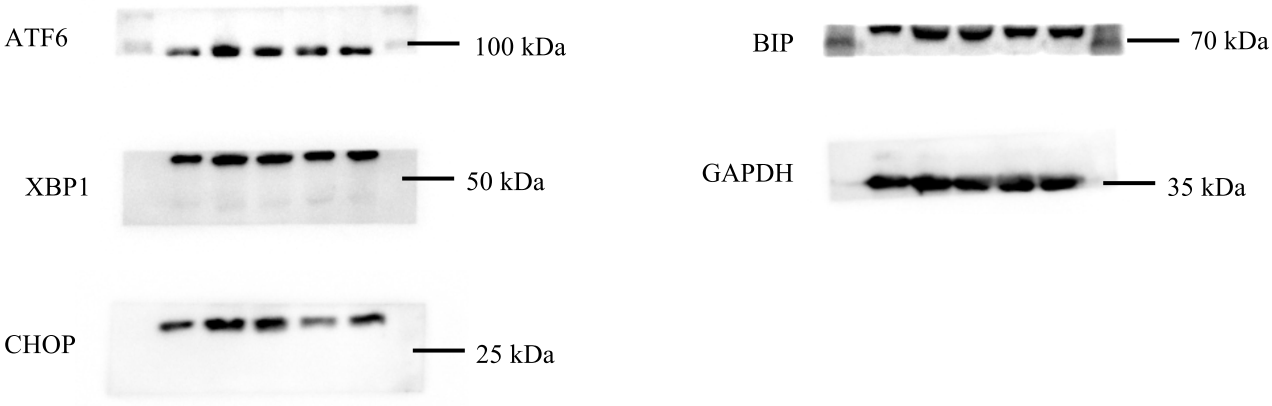

Repeat 2

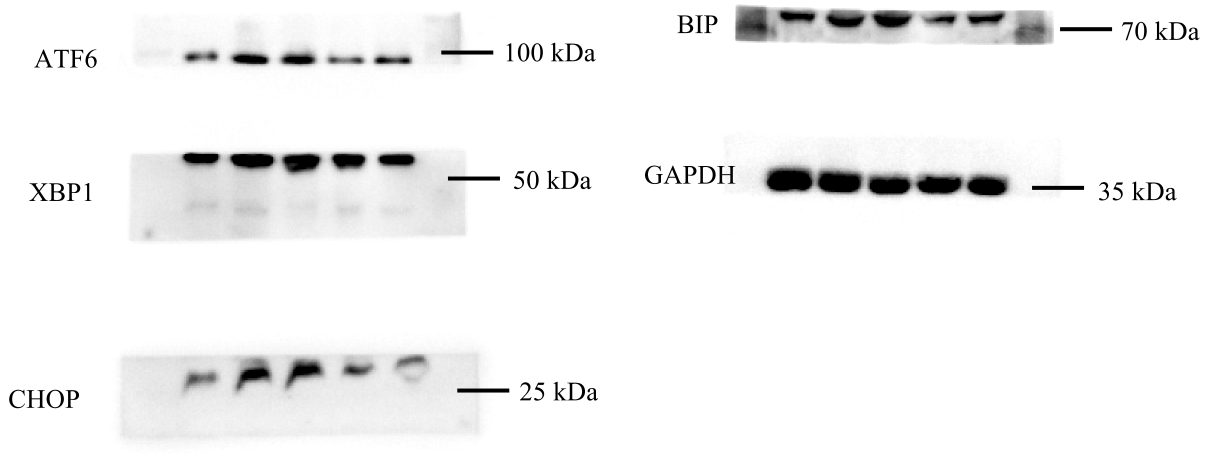

Repeat 3

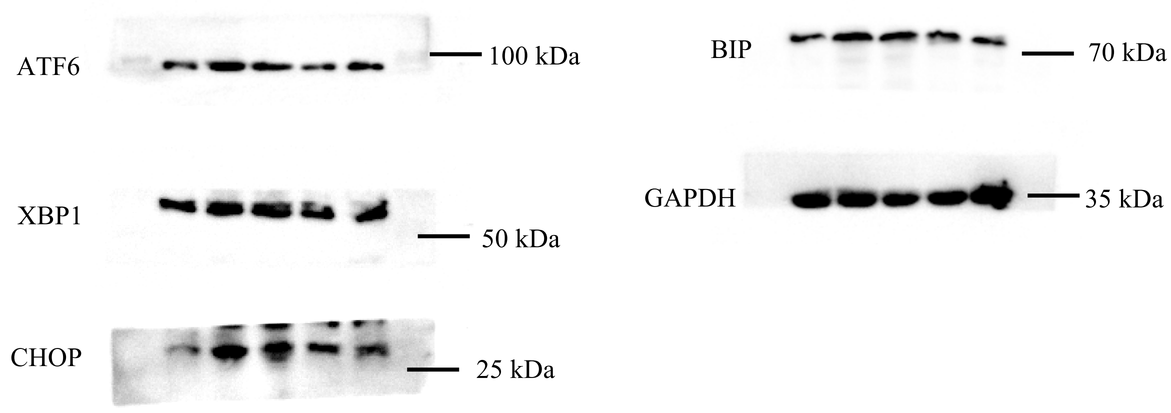

Figure 7C

Repeat 1

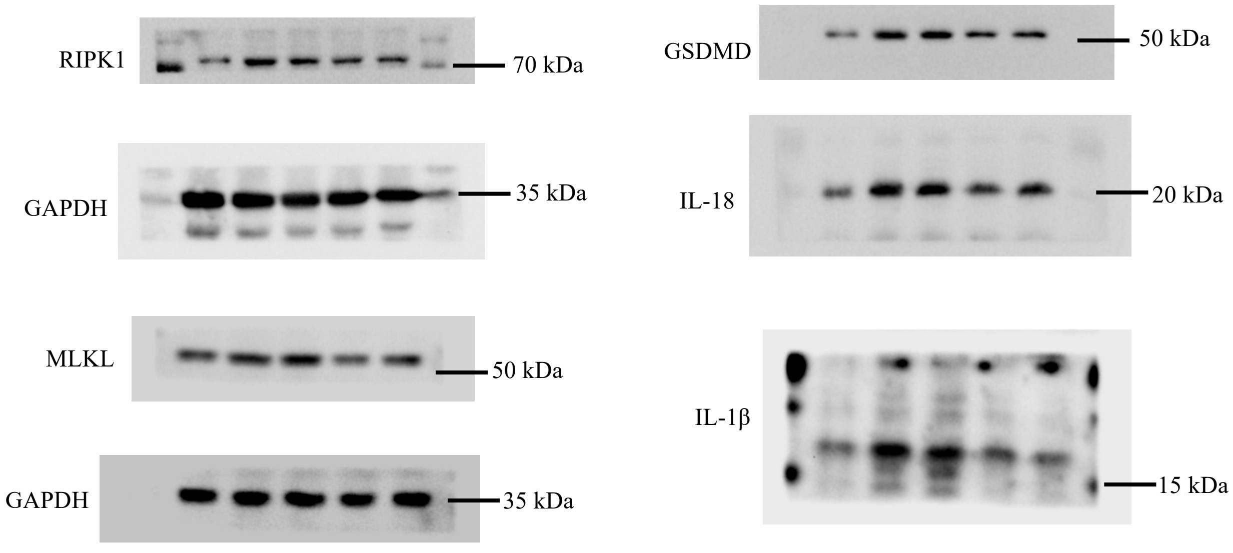

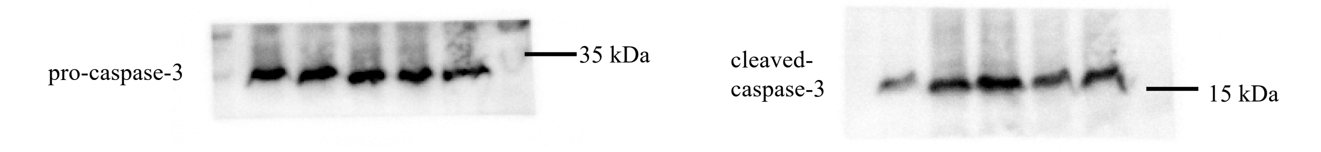

Repeat 2

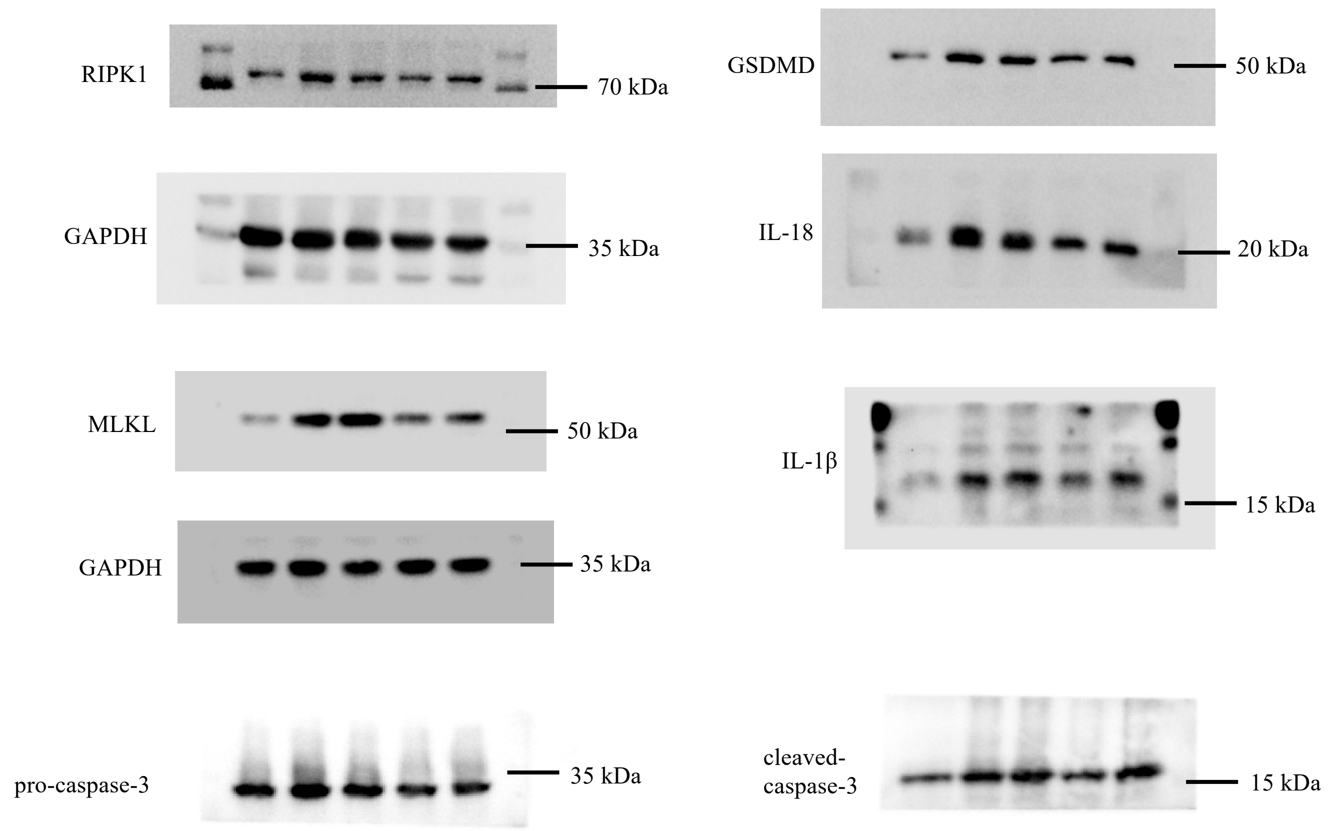

Repeat 3

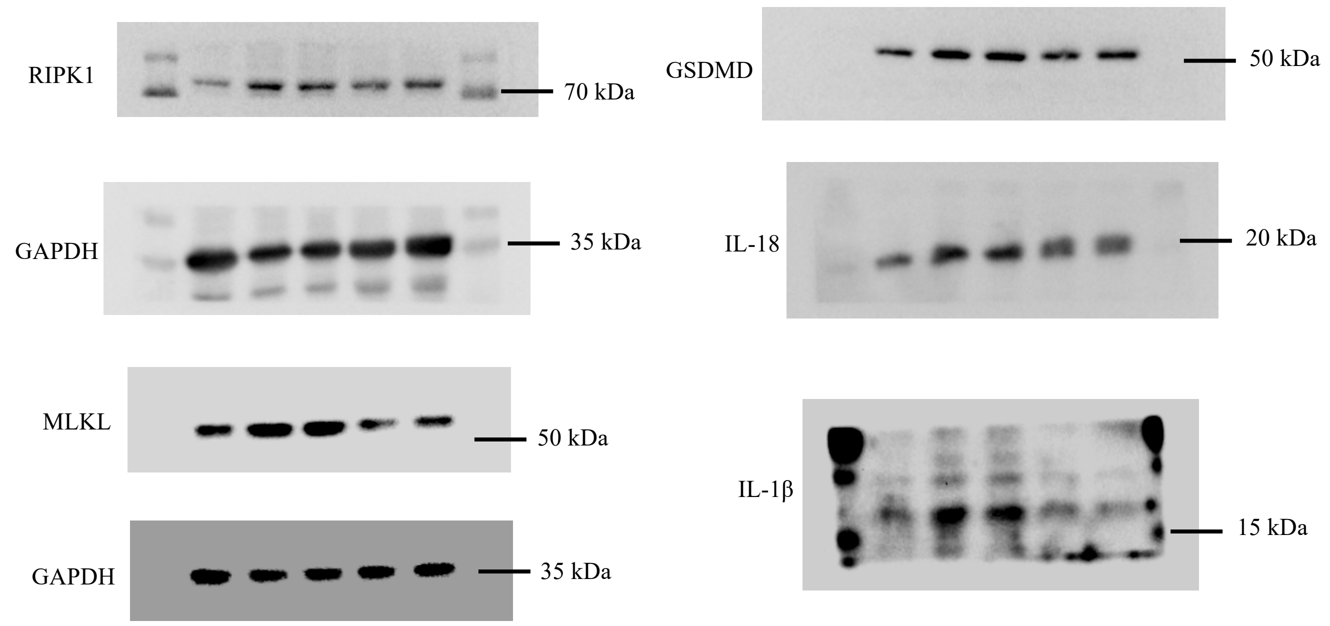

pro-caspase-3

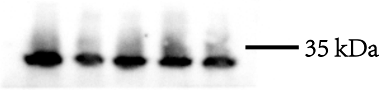

— 35 kDa

cleaved-caspase-3

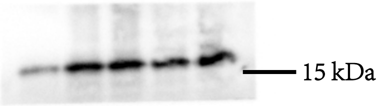

— 15 kDa
